# The global cost of schizophrenia: a health economic modelling study

**DOI:** 10.1186/s13033-026-00740-x

**Published:** 2026-09-29

**Authors:** Luisa S. Welter, Mateo Montes-Martínez, Lara Bohr, Marjan Arvandi, Nikolai Mühlberger, Paul Boon, Günther Deuschl, Claudio L. A. Bassetti, Thomas Berger, Thomas G. Schulze, David H. V. Vogel, Alexandra Philipsen, Miriam P. L. John, Uwe Siebert, Richard Dodel, Frank Jessen, Peter Falkai

**Affiliations:** 1 Department of Geriatric Medicine, University Duisburg-Essen, Essen, Germany; 2 Department of Health Services Research, Division Health Economics, School of Medicine and Health Sciences, Carl von Ossietzky University Oldenburg, Oldenburg, Germany; 3 Department of Psychiatry and Psychotherapy, Faculty of Medicine and University Hospital Cologne, University of Cologne, Cologne, Germany; 4 Department of Public Health, Institute of Public Health, Medical Decision Making and Health Technology Assessment, Health Services Research and Health Technology Assessment, UMIT TIROL - University for Health Sciences and Technology, Hall in Tirol, Austria; 5 Department of Neurology, 4Brain Ghent University Hospital & Ghent University, Ghent, Belgium; 6 Department of Neurology, Universitäts-Klinikum Schleswig-Holstein, Campus Kiel, Christian-Albrechts-University, Kiel, Germany; 7 Neurology Department, Inselspital, University Hospital, University of Bern, Bern, Switzerland; 8 Department of Neurology, Medical University of Vienna, Vienna, Austria; 9 Comprehensive Center for Clinical Neurosciences & Mental Health, Medical University of Vienna, Vienna, Austria; 10 Institute of Psychiatric Phenomics and Genomics (IPPG), University Hospital, Ludwig Maximilian University of Munich, Munich, Germany; 11 Department of Psychiatry and Psychotherapy, University Hospital Bonn, Bonn, Germany; 12 Department of Psychiatry, Ludwig-Maximilians-University Munich, Munich, Germany; 13 Max-Planck-Institut of Psychiatry, Munich, Germany; 14 Center for Health Decision Science and Departments of Epidemiology and Health Policy & Management, Harvard T.H. Chan School of Public Health, Boston, MA USA; 15 Center for Health Technology Assessment, Mass General Brigham, Harvard Medical School, Boston, MA USA; 16 German Center for Neurodegenerative Disease (DZNE), Bonn, Germany

**Keywords:** Schizophrenia, Psychotic disorders, Cost of illness, Disease burden, Psychiatry

## Abstract

**Background:**

Schizophrenia is a chronic psychiatric disorder that leads to substantial disability, impaired quality of life, and reduced life expectancy. In addition to its profound impact on affected individuals, the disorder places a considerable financial burden on health care systems, caregivers, and society. Although the cost of schizophrenia has been studied in several individual countries, comprehensive global estimates across multiple cost categories remain scarce. This study aimed to estimate the global socioeconomic burden of schizophrenia using health economic modelling.

**Methods:**

We conducted a prevalence-based cost-of-illness analysis for 204 countries in 2019, focusing on adults aged 20 years and older. Data on direct (medical and nonmedical), indirect (productivity losses), and informal (nonprofessional, unpaid) care costs were systematically retrieved through a PubMed-based literature search covering records published between 02/2010 and 04/2023. Extracted per-patient costs were standardized to 2019 purchasing power parity (PPP) USD, and missing data were imputed using a stratification approach based on four gross national income (GNI) groups. Costs were scaled to the national level by multiplying the per-patient costs by country-specific prevalence estimates from the Global Burden of Disease Study. Costs were additionally expressed as a proportion of gross domestic product (GDP). To ensure data validity from a clinical perspective, all estimates were scrutinized by a panel of psychiatry experts.

**Results:**

An estimated 22.1 million adults were living with schizophrenia in 2019 (0.39% prevalence). Total economic costs across upper-middle, lower-middle, and high-income regions reached $243.2 billion (PPP), with direct costs representing 47%, indirect costs 29%, and informal care costs 24%. Upper-middle-income countries bore the greatest burden relative to GDP, whereas high-income countries had the highest per-patient and national costs.

**Conclusions:**

This study provides the most comprehensive global assessment to date of the economic burden of schizophrenia. Per-patient costs were identified as the main driver of the absolute global burden, underscoring the importance of effective disease management strategies. Policy-makers and other stakeholders should prioritize interventions that enhance patient functioning and societal participation.

**Supplementary Information:**

The online version contains supplementary material available at https://doi.org/10.1186/s13033-026-00740-x.

## Introduction

Schizophrenia is a chronic psychiatric disorder and ranks among the most disabling conditions across medical specialties [1]. Core symptoms include delusional and disorganized thinking as well as hallucinations [2]. Prominent negative symptoms such as impoverished motivation (avolition) and anhedonia contribute to social withdrawal and markedly impair quality of life [3, 4].

Schizophrenia affects approximately 0.35 of the general population at any given point in time, with an estimated lifetime prevalence of 0.62% to 0.64% [5, 6]. Life expectancy is critically reduced by an average of 15 years, compared with that of the general population [7], a gap attributable to factors such as suicide, accidental death, and comorbid medical conditions [8]. Importantly, a portion of this excess early mortality may be preventable through timely and adequate treatment [9]. Both pharmacological treatment and psychosocial interventions are essential components of effective disease management and are critical for improving patient outcomes.

Beyond the burden experienced by patients themselves, schizophrenia exerts substantial effects on society at large. Those affected are frequently hindered in their ability to participate fully in their community life and typically require intense medical care and social support. To illustrate, employment rates among persons with schizophrenia are considerably lower than those among the general population [10], and health care utilization, specifically hospitalization, is much greater [11, 12]. Moreover, schizophrenia places a significant strain on informal caregivers, with psychological (e.g., stress), physical, and financial consequences [13–15]. In regions where access to psychiatric services is limited, reliance on family members for caregiving becomes even more pronounced [16]. Consequently, the economic burden arises from direct health care and social care expenditures (direct costs), productivity losses due to reduced labour participation (indirect costs), and financial losses associated with informal caregiving (informal care costs).

Most cost-of-illness analyses on schizophrenia have focused on individual countries or on specific cost components, and recent reviews and meta-analyses have summarized existing primary evidence on the costs of schizophrenia, characterized methodological approaches, and noted the uneven availability of data across income regions [17–21]. For instance, Lin et al. (2023) reported annual societal per-patient costs ranging from US$819 (Nigeria) to US$94,587 (Norway), and Imre et al. (2026) documented median annual per-patient costs of $33,236 (mean $47,872) [19, 20]. Both systematic literature reviews underscore the scarcity of cost data in low- and middle-income regions. Modelling approaches can help to bridge these gaps in primary research even where primary data are unavailable. Mitchell et al., for instance, analysed healthcare expenditure across 204 countries and found that although schizophrenia has a comparatively low prevalence, it accounted for over 11% of global healthcare spending on mental disorders in 2019. However, no recent study has combined multiple cost domains with global regional coverage while also imputing estimates for regions lacking primary data.

The present study aimed to address this research gap through a novel health economic modelling and imputation approach that groups countries by gross national income (GNI) group, enabling cost estimates to be derived for data-scarce countries by combining evidence from economically comparable nations while accounting for country-specific economic parameters.

This research is part of broader initiative to assess the worldwide societal costs of brain (mental and neurological) disorders, facilitating comparability across diseases through the implementation of a shared health economic modelling framework.

## Methods

This study aimed to assess the annual direct, indirect, and informal care costs of schizophrenia across 204 countries worldwide. The study focused on the adult population (20 years and older). Minors were excluded, as they are typically not considered of working age and may often still reside with their parents, factors likely to produce care and cost dynamics distinct from those in later life. The higher age threshold of 20 years, rather than 18 years, was determined by the data source, which did not provide pooled estimates for the population aged 18 years or older [22]. This cut-off also ensures comparability with other disorders examined within the broader research initiative of which this study forms part. Given the incomplete availability of country-specific economic data for the period following the COVID-19 pandemic (2020–2023) and to avoid distortions in typical health care utilization patterns during the pandemic years, 2019 was selected as the index year.

Building on the COIN-Eu methodology (Siebert et al., [51]), the analytical approach employed four key steps. First, schizophrenia prevalence data for 2019 were retrieved from the Global Burden of Disease (GBD) Study 2021 [23]. Second, a systematic literature search was conducted to identify appropriate inputs for the economic model, considering data on direct, indirect, and informal care costs. Third, data synthesis and imputation were performed to address missing information. Fourth, the model’s outputs were reviewed by a panel of clinical experts to ensure plausibility.

### Included cost components

Drawing on multiple payer perspectives, costs were grouped into three broad categories: direct (medical and nonmedical), indirect, and informal care costs. Direct costs encompassed all medical expenditures associated with diagnostic and therapeutic procedures, including inpatient and outpatient care and drug costs, as well as nonmedical services such as social services and assisted living costs. Indirect costs were defined as productivity losses, including those related to premature mortality and productivity losses (absenteeism and presenteeism). Informal care costs represented the monetary value of unpaid, nonprofessional caregiving provided by partners, relatives, or other informal caregivers. Direct and indirect costs represent the conventional categorisation in cost-of-illness research on schizophrenia, with informal care costs frequently omitted or subsumed under indirect costs. We chose to treat informal care costs as a distinct domain for three reasons. First, prior research indicates that the informal care burden in schizophrenia can be substantial and has significant financial ramifications [13, 24]. Second, the policy implications of the three cost domains differ considerably, justifying their analytical separation. Third, estimating informal care costs requires methods that differ from those used for direct non-medical and indirect costs [25], and combining them would obscure important methodological distinctions. We did not include cost elements related to judicial or legal costs, tax revenue losses, or unemployment costs. A detailed breakdown of the specific cost components included within each cost category is provided in Supplementary Table 1.

### Retrieval of prevalence data

Prevalence and population size data for each country were obtained from the Global Burden of Disease Study 2021 via its Results Tool [22] for adults of both sexes aged 20 years and older. To estimate indirect costs related to labour-market productivity losses, corresponding prevalence data for the working-age population (20–64 years) were also retrieved.

### Identification of economic data

Systematic searches were conducted in PubMed to identify country-specific, annual, and per-patient cost estimates. The search algorithm was previously developed and tested during a pilot phase by LW, LB, and MMM. The final search strategy combined disease-specific and economic terminology using MeSH terms and title-based keywords and included articles published between February 2010 and April 2023. The full search query is documented in Table 2 in the Supplementary Material.

Titles, abstracts, and full texts were subsequently screened for eligibility by LB and LW2* according to the predefined criteria outlined in Supplementary Table 3. Studies employing various cost assessment approaches—including bottom-up and top-down methods—were considered, irrespective of the payer perspective (e.g., health care system, employer, or patient). A key inclusion criterion was the documentation of cost data of at least one cost (sub)component. Articles limited to narrowly defined cost subtypes (e.g., exclusively hospitalization costs) were excluded (for more details, see Supplementary Table 1). Similarly, studies that merely reported overall societal costs without further disaggregation were also excluded. To ensure a contemporary cost perspective, only prevalence-based reports were included, whereas lifetime cost analyses (i.e., incidence-based studies) were excluded because of their reliance on long-term modelling assumptions.

Data were extracted in parallel by two researchers (LB and LW2*) into a matrix originally developed for the COIN-Eu study; the extracted variables included country, publication details, cost year, currency with or without purchasing power parity (PPP) adjustment, and main cost components (direct, indirect, and informal care), along with their respective subcategories. The extraction template, which also served as a protocol for subsequent quality checks, is provided as Supplementary Table 4.

When the reported data did not align with the required per-patient, annual format, appropriate conversion steps were applied. For example, when only national-level cost estimates were available, per-patient estimates were calculated by dividing by disease prevalence. If prevalence was not reported within the economic study, estimates from the GBD prevalence data were used. All included sources and decisions made during the extraction process are documented in Table 6 in the Supplementary Material. Following the initial data extraction, each data point was independently cross-checked by LW. Discrepancies were discussed during in-person and online meetings between LB, LW2*, LW, and MMM, which led to the exclusion of some papers deemed methodologically unsuitable, for instance, due to sampling biases or a very narrow scope of cost reporting (for more details, see Supplementary Table 1).

### Data synthesis and imputation

Data harmonization and imputation steps follow the methodology described by Siebert et al. [51]. To account for heterogeneity in the primary studies that reported costs for different years and currencies, the extracted annual per-patient costs were standardized to PPP-adjusted U.S. dollars (USD) 2019. Cost data expressed in national currencies were first adjusted for inflation using the World Bank Consumer Price Index (CPI) [26], and then converted to USD using PPP from the World Bank and the Organization for Economic Cooperation and Development (OECD) [27].

Data synthesis largely used a median-based approach, reducing the impact of extreme values. First, in cases where studies reported only direct medical costs, but not direct non-medical or aggregated total direct costs, non-medical costs were imputed using the median value from other studies conducted in the same country, where such data were available. These imputed non-medical costs were then added to the direct medical costs to calculate total direct costs. For countries with more than one record, the median was calculated per cost category.

To address countries lacking primary cost data, an imputation procedure adapted from Begley et al. was applied [28]. This method incorporates the differing economic contexts by classifying countries as belonging to one of four gross national income (GNI) groups, as defined by the World Bank: low-income, lower-middle-income, upper-middle-income, and high-income. Median values were calculated within each GNI group and for each cost type (direct, indirect, and informal care) based on countries with available data. Country-specific cost estimates were then derived by weighting these medians using economic parameters from the World Bank (2019). Specifically, for the imputation of total direct and informal care costs, a weighting factor was applied based on the ratio of a country’s health expenditure (as a percentage of GDP) to the corresponding average for all countries within the same GNI group, derived from World Bank data for 2019 [29]. For the imputation of indirect and informal care costs, weighting was based on the ratio of a country’s GDP per capita to the average GDP per capita of its GNI group, likewise derived from World Bank data for 2019 [30]. Supplementary Table 8 documents these economic parameters per country. This approach produced imputed values for countries lacking primary data when data from another country within the same GNI group were available. A few exceptions were countries (e.g., Venezuela) for which the necessary model parameters were not available at the time of analysis.

Per-patient costs (imputed or non-imputed) were subsequently scaled to the national level by multiplying by country-specific prevalence: the adult population (≥ 20 years) for direct and informal care costs and the working-age population (20–64 years) for indirect costs. To contextualize economic burden relative to national economic resources, supplementary analyses expressed costs as a percentage of each country’s 2019 GDP [26].

### Expert data validation

Four board-certified psychiatry experts (TS, DV, MJ, JS*) evaluated the plausibility of the cost estimates from a clinical perspective following a predefined four-step appraisal procedure (for method details, see Siebert et al., [51]). Both original and imputed schizophrenia cost estimates were assessed at the patient and national levels. Psychiatry experts independently completed their appraisals using written instructions, an Excel document containing all preliminary data visualised as bar charts, and a template for documenting their comments (see Supplementary Fig. 1 for the instructions and template). Where necessary, outstanding questions regarding the approach were resolved through online meetings with the extraction team (MMM, LW, LB, LW2*). Across the psychiatry experts, 20 extracted data points from 17 primary studies were flagged as being of uncertain plausibility. The underlying sources were re-examined by the data extraction team and senior psychiatrist JF to assess extraction accuracy and methodological suitability. Following consensus, one study was excluded due to an incidence-based design, another one due to an overly narrow cost assessment, and six data points were revised.

### Regression analysis

To explore associations between societal costs, economic status, and disease burden, we conducted a supplementary multiple linear regression analysis. Societal costs were operationalised as total national costs (USD 2019), economic status as GDP (USD 2019), and disease burden as the total number of prevalent schizophrenia cases in 2019. Societal costs per country were specified as the dependent variable, while GDP and prevalence were entered as independent variables. Prior to the analysis, all variables were standardised to z-scores based on their respective means and standard deviations. Supplementary Table 9 presents the non-standardised data used for the regression analysis, including societal costs, GDP, and absolute schizophrenia prevalence for each country.

Given that this study relied exclusively on publicly available data and did not involve individual patient information, it was exempt from ethical approval requirements.

## Results

### Study selection

The PubMed-based search strategy yielded 657 unique articles, of which 46 met the eligibility criteria for inclusion in the health economic analysis. The selected studies were unevenly distributed across income groups: the majority were from high-income regions (*n* = 31, 67%), followed by upper-middle-income regions (*n* = 13, 28%) and lower-middle-income regions (*n* = 2, 4%). No eligible primary cost analyses were identified from low-income countries. The record selection process is documented in a PRISMA flowchart, provided Supplementary Fig. 2.

From the 46 included studies, 85 unique data points were extracted. Nearly half of these focused on direct medical costs (*n* = 39, 46%). The distribution of data points by cost category and income group is shown in Table 5 in the Supplementary Material. Supplementary Table 7 provides an overview of key methodological characteristics of the included studies.

### Prevalence

**Fig. 1:**
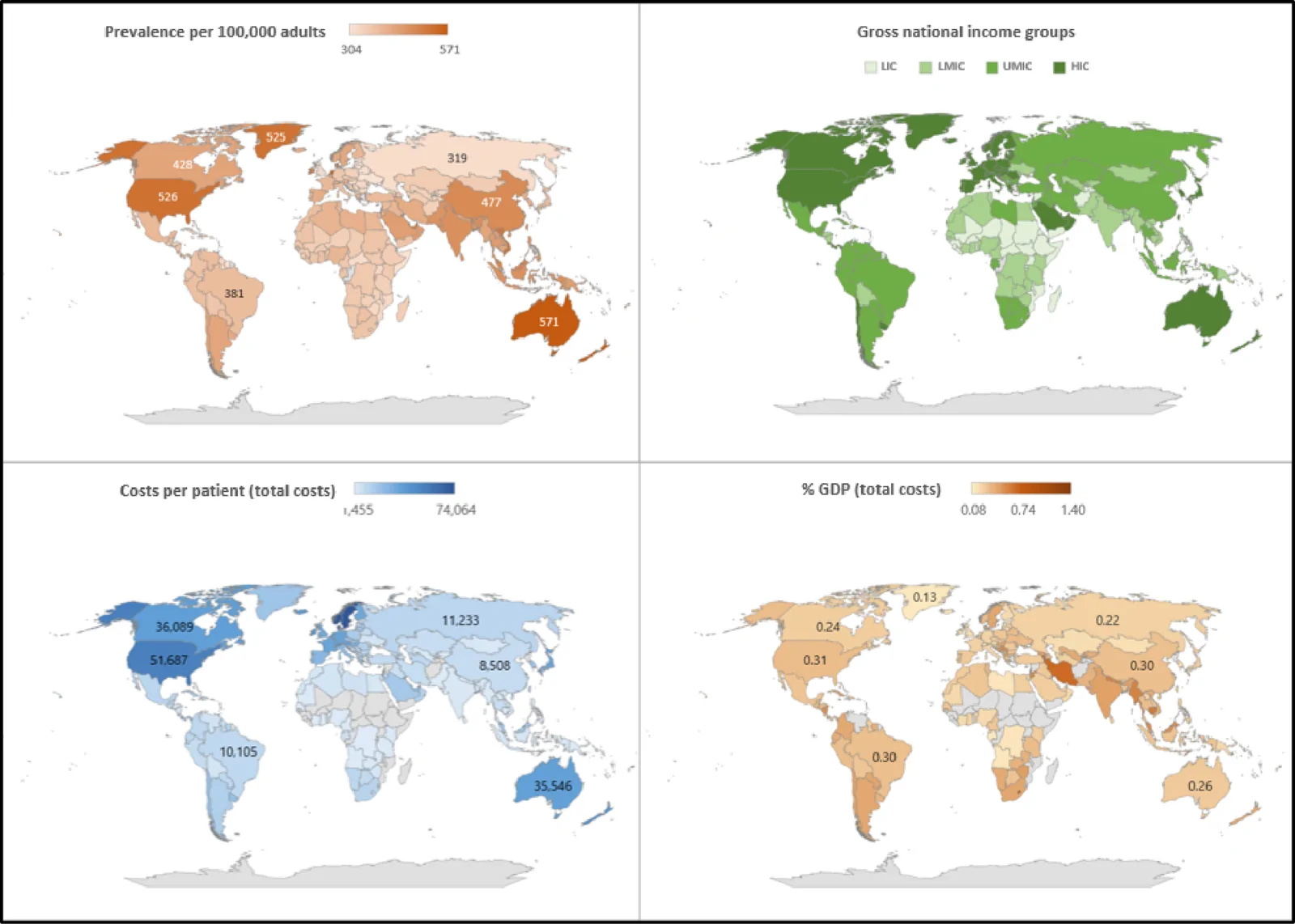
Prevalence and costs of schizophrenia. Choropleth showing schizophrenia prevalence per 100,000 adults in 2019 (upper left), country classification into four national income groups (upper right), total annual costs per patient expressed as 2019 USD, PPP (lower right), and total annual national costs expressed as a percentage of GDP (lower right). Grey indicates that no viable primary or imputed data were available for these countries. Abbreviations: LIC: low-income countries, LMIC: lower-middle-income countries, UMIC: upper-middle-income countries, HIC: high-income countries, GDP: gross domestic product

In 2019, the global prevalence of schizophrenia was estimated 22.1 million affected adults. Given the global population of more than 5 billion adults, this equates to a mean prevalence of 389 per 100,000 adults (0,39%). The highest regional prevalence was observed in high-income countries (415 per 100,000), followed by lower-middle-income regions (387 per 100,000). Upper-middle-income (379 per 100,000) and low-income (347 per 100,000) regions ranked third and fourth, respectively. Figure 1 (upper left side) illustrates prevalence rates across the globe on a Mercator map.

### Costs

#### Total societal costs

Across the three income regions combined (LMIC, UMIC, and HIC), the total global economic burden in 2019 was estimated at $243,158 million (PPP). 47% (47%) of this amount was attributed to direct costs, 24% to informal care costs, and 29% to indirect costs.

**Fig. 2:**
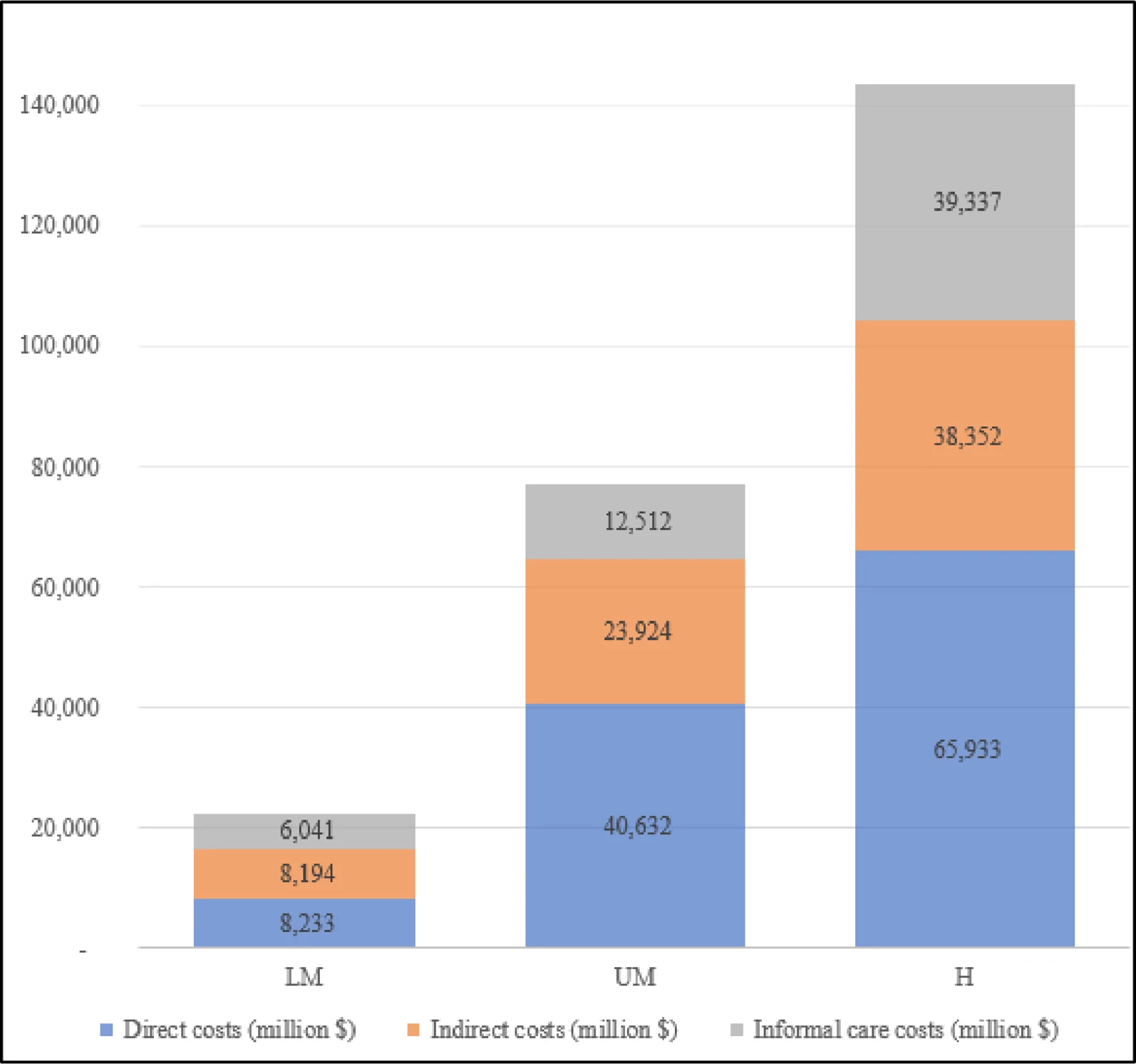
Costs of schizophrenia per national income group and cost category. Total direct, indirect, and informal care costs per national income group in 2019 (million USD PPP)

High-income regions, comprising 66 countries and an adult population of approximately 960 million, accounted for by far the largest share of the aggregate economic burden, with total costs of $143,623 million (PPP). Upper-middle-income regions, including 55 countries and a substantially larger adult population of 2,089 million, contributed the second-highest total costs, estimated at $77,068 million (PPP). Lower-middle-income regions, encompassing 50 countries and 1,765 million adults, incurred the lowest overall costs, totalling $22,468 million (PPP) (Fig. 2).

In lower-middle-income regions, the cost components were relatively evenly distributed, with direct costs accounting for 37% of the total cost, indirect costs for 36%, and informal care costs for 27%. In upper-middle-income regions, direct costs emerged as the dominant component (53%), followed by indirect costs (31%), while informal care costs constituted a smaller proportion (16%). In high-income regions, direct costs also emerged as the largest cost category (46%), whereas informal care and indirect costs constituted equal proportions, each accounting for 27% of the total costs.

The total national costs per country are detailed in Supplementary Table 7. This table also provides a breakdown of direct, indirect, and informal care costs at the national level, indicating where data were imputed. Since the total national costs differ primarily because of population size, further statistical comparisons at the country level are not given here.

#### Costs per patient

The total per-patient costs varied substantially across income regions (Fig. 1, lower left side). The highest costs were observed in high-income countries, ranging from 8,477 in the Northern Mariana Islands to 74,064 in Norway (median: 23,031; mean: 27,507; SD: 14,71). In comparison, per-patient costs were considerably lower in upper-middle-income countries, ranging from 3,846 in Indonesia to 25,191 in Tuvalu (median: 9,536; mean: 10,103; SD: 3,56). By far, lower-middle-income regions had the lowest per-patient costs, from 1,455 in Palestine to 6,634 in Micronesia (median: 3,086; mean: 3,254; SD: 1,13). Country-specific per-patient costs (direct, indirect, and informal care) are detailed in Table 7 in the Supplementary Material.

#### Cost relative to GDP

Compared with national income groups, costs accounted for the largest share of economic output in upper-middle-income countries, ranging from 0.13% (Gabon) to 1.40% (Tuvalu) of GDP (median: 0.31; mean: 0.36%; SD: 0.22). In lower-middle-income countries, costs relative to GDP were slightly lower and with less variation between countries, ranging from 0.08% (Palestine) to 0.79% (Kiribati) (median: 0.27; mean: 0.32%; SD: 0.15). Schizophrenia costs as a proportion of GDP were smallest in high-income countries, ranging from 0.09% (Puerto Rico) to 0.67% (Palau) (median: 0.23; mean: 0.24%; SD: 0.10). Here, the variability was also the lowest. Figure 1 (lower right side) shows total national costs relative to GDP on a Mercator world map. For a graphical summary of data variability, boxplot diagrams are provided in the Supplementary Material Fig. 3.

#### Relationships between societal costs, GDP, and prevalence

The supplementary multiple linear regression analysis showed a strong association between societal costs, economic status (expressed as GDP), and disease burden (approximated by prevalence), with the model accounting for 99% of the variance in total societal costs (R² = 0.99, F(2, 200) = 9826.05, *p* < .001). GDP was strongly positively associated with total societal costs (β = 0.967, *p* < .001). Specifically, the coefficient indicates that a one-standard-deviation increase in GDP was associated with a 0.967-standard-deviation increase in total societal costs, independent of absolute schizophrenia prevalence. Schizophrenia prevalence also showed a positive but comparatively small independent association with total societal costs (β = 0.043, *p* < .001). Key regression coefficients and model statistics are reported below Supplementary Table 9. These findings indicate that variation in societal costs was substantially more strongly associated with variation in GDP than with variation in absolute schizophrenia prevalence.

## Discussion

This cost-of-illness study provides comprehensive estimates of the global economic burden of schizophrenia, covering 171 high- and middle-income countries and territories. To our knowledge, this is the first analysis to systematically address gaps in the primary literature at a global level using imputation, enabling direct comparisons across national income regions, countries, and cost categories, including direct, indirect, and informal care costs.

The global cost of schizophrenia was estimated at $243,158 million (PPP) in 2019. Despite the relatively low prevalence of approximately 0.4% in the adult population, schizophrenia imposes a substantial financial strain on individuals, health care systems, and societies. Nearly half (47%) of the total costs were direct costs, including medical expenditures and social services. Less than one-third (29%) were indirect costs from reduced workforce participation, and one-fifth (24%) reflected financial losses associated with informal caregiving. Although the magnitude of informal care costs may appear high from a patient-centred perspective, it is consistent with the substantial caregiver burden documented in other works [13–15]. Importantly, informal care is often not reported as a stand-alone cost category in cost-of-illness research on schizophrenia [19]. Given its substantial contribution to total costs, however, it is beneficial not to conflate them with other cost components. Treating informal care as a distinct category has notable policy implications, as the resulting need for caregiver support is a fundamentally different concern from the need to improve labour market integration, which relates to workforce losses, or the need for more effective treatments, which relates more closely to direct costs.

The relative contribution of different cost types shows notable heterogeneity across the literature [19], often stemming from differences in how cost types are categorised and labelled. For instance, a review by Chong et al. (2016) found that indirect costs accounted for 50%–85% of total schizophrenia costs [31], a considerably higher proportion than observed in the present study. This discrepancy is partly explained by the inclusion of costs that, under the framework applied here, would be classified as informal care costs, such as early retirement among caregivers. Furthermore, our study did not capture legal costs, such as incarceration, or unemployment costs (see limitations), which further accounts for the difference. Yet other reviews categorizing unemployment and informal care costs as indirect costs report cost dynamics in which direct and indirect costs contribute roughly equally [20]. Methodological differences in cost accounting also play an important role. Studies that capture only expenses directly attributable to schizophrenia treatment will yield lower estimates than those contrasting the costs of patients with schizophrenia and individuals without the disorder. The latter approach additionally captures costs arising from comorbidities, sometimes referred to as intangible costs [32], leading to higher cost estimates. Greater standardisation in cost categorisation and accounting methods would substantially improve the comparability of future research in this area.

This study reveals that regional cost disparities differ depending on the analytical perspective applied. While high-income countries incur the highest absolute per-patient and total national costs, upper-middle-income regions bear the greatest relative economic burden when costs are expressed as a proportion of GDP. Relatedly, our findings highlight a structural mismatch between economic burden (that is, costs relative to GDP) and the extent of scientific investigation. This analysis thereby confirms previous observations that cost-of-illness research on schizophrenia has been overwhelmingly concentrated in high-income settings [19, 31]. Data availability declines markedly with national income, and owing to a lack of primary studies, no estimate could be generated for low-income countries in the present analysis. Although evidence from middle-income regions remains limited, our imputation approach enabled the generation of best current estimates for countries lacking original cost data, thereby partially addressing this evidence gap.

Another interesting finding is that the relative contribution of economic components (i.e., direct, indirect, and informal care costs) varied substantially across regions. These differences may in part reflect regional disparities in access to health care services, care delivery structures, and data availability in the literature. For instance, the increasing share of direct costs from lower-middle- to upper-middle-income regions may indicate improved availability and utilization of formal health services [33]. In contrast, the comparatively high contribution of informal care costs observed in high-income regions is surprising and warrants further examination. Given that higher national income is generally associated with better health care quality and access [33] and thus, reduced reliance on informal care, one would expect informal care costs to be lowest in high-income regions. In fact, research indicates that caregiver burdens are 2 to 3-fold higher in low and lower-middle-income countries compared to higher-income countries [34]. This pattern was not observed in our estimates, suggesting that additional factors—such as differences in valuation methods or reporting practices—may influence the observed distribution of cost components. Methodological heterogeneity in cost reporting further complicates the definitive interpretation of these patterns. With regard to absolute (per-patient) costs, differences between income regions reflect, among other factors, varying price levels and average salaries.

Finally, our supplementary exploratory analysis suggests a strong association between societal costs and GDP, whereas disease burden, expressed as prevalence, showed a comparatively smaller association with societal costs. This finding is consistent with the observation that per-patient costs tend to be higher in higher national income groups, with mean costs per patient being more than sevenfold higher in high-income countries than in lower-middle-income countries ($23,031 versus $3,086 PPP, 2019, respectively). As noted above, several real-world factors may help explain this pattern, including differences in access to healthcare, variations in treatment prices and average wages, as well as methodological reasons related to the better-documented costs in high-income regions, which the current study also highlights. However, the findings from this exploratory analysis should be interpreted with caution, as both GDP and prevalence were input parameters of the cost estimation model, and primary cost data were limited. Further research is therefore warranted to better understand the key drivers of societal costs.

Our findings are broadly consistent with those of previous cost-of-illness analyses of schizophrenia and other psychotic disorders. Gustavsson and colleagues estimated the cost of psychotic disorders in Europe in 2010, addressing gaps in the primary literature through imputation, similar to the current analysis [35]. The authors reported a higher prevalence of schizophrenia (0.64% in the adult population) than the GBD data utilized in the current study. Total per-patient costs were estimated at €18,796 (PPP, 2010). These older data were utilized in a recent modelling study that inflated 2010 values to 2019 and added an alternative approach to estimate indirect costs, namely, the willingness-to-pay (WTP) framework, which assigns a theoretical monetary value to DALYs [36]. The 2019 total (direct and indirect) cost per patient with schizophrenia in Europe was estimated at €21,424 using the human capital method and €27,495 using the WTP method. These findings are similar to our median per-patient cost in high-income regions of $23,031 (PPP, 2019).

A recent global modelling study by Mitchell et al. estimated direct health care spending for schizophrenia in 2019 at $94.5 billion, corresponding to $3,698 per case worldwide (2021 USD) [37]. Although our analysis adopts a broader definition of direct costs—incorporating both medical and nonmedical expenditures—our findings are broadly comparable. We estimate total direct costs of approximately $115 billion across three global income regions, with a median per-patient cost of $4,455 (PPP, 2019). This indicates a notable degree of consistency across studies despite differences in cost definitions, valuation approaches, and analytical frameworks.

Alongside imputation-based approaches, several reviews have investigated the economic burden of schizophrenia globally and across larger geographical regions. A recently published umbrella review and meta-analysis by Imre et al. (2026), synthesizing 26 systematic reviews, reported median annual per-patient direct costs of $23,126 (medical: $19,543; ancillary: $1,152) and indirect costs for $21,333 (all expressed in 2024 USD) [20]. In line with the current study and other reports, the authors emphasize substantial cost gradients across income levels, with median total cost of $34,175 in high-income regions, $3,345 in upper-middle-income regions, and $3,452 in lower-middle-income regions. Overall, review-based cost estimates tend to exceed those reported in the present study. For instance, the median total annual cost per patient reported by Imre et al. (2026) was $33,236, compared with $10,105 in the current study. Beyond differences in cost type selection (as discussed above and further elaborated in the Limitations section), an additional explanation may lie in the composition of the underlying data: medians derived from reviews are, even when stratified by income group, likely skewed towards more resourceful countries with greater research output. In contrast, the medians reported here incorporate countries for which no primary data exist, thereby reflecting a broader pool of countries.

A recurring finding across reviews [19, 20], and confirmed by the present study, is the scarcity of cost-of-illness research in low- and middle-income regions. This gap is particularly consequential for policymakers in these countries, who lack the data needed to guide decision-making, as findings from economically dissimilar settings cannot readily be transferred across contexts. While not a substitute for primary research, imputed estimates such as those presented here can provide a preliminary indication of costs until country-specific data become available.

Beyond financial costs, schizophrenia constituted nearly 10% (9.53%) of all disability-adjusted life years (DALYs) attributable to mental disorders in 2021 [38]. Taken together, these findings further underscore that the burden of schizophrenia is driven less by its population prevalence and more by high levels of disability and intensive care requirements at the individual level.

### Implications

Prevalence, incidence, and DALYs attributable to schizophrenia are projected to rise through 2035 [39], highlighting the need for effective mid- and long-term strategies for disease management. The sizable indirect and informal care costs imply persistent gaps in the adequacy and continuity of current health care provisions for individuals with schizophrenia. Evidence-based prevention and therapeutic interventions, particularly those emphasizing early detection and timely treatment, have the potential to generate considerable returns on investment by mitigating long-term disability and associated societal costs. Consistent with this, the European Brain Council has defined several key action points, including better treatment access for negative symptoms and cognitive impairment that drive functional disability and are insufficiently treated, or even aggravated, by current antipsychotics [40].

Although genetic liability accounts for 70–80% of schizophrenia risk, its aetiology is best conceptualized within a diathesis-stress model [41]. Psychosocial factors, such as social isolation and childhood trauma, interact with inherited vulnerability and other biological stressors to contribute to disease pathogenesis [42–44]. Therefore, targeting modifiable stressors during sensitive periods may reduce onset risk, symptom severity and/or adversity, and, consequently, societal costs. After diagnosis, in addition to pharmacotherapy, psychosocial interventions, such as supported employment and community support, can improve functional outcomes and facilitate social and occupational (re-)integration [40, 45–47]. Such interventions may also lower the economic burden to health care systems, social security, and overall society by reducing unemployment rates, decreasing recurrent hospitalizations, and reducing reliance on both formal health care services and informal care.

### Limitations

This cost-of-illness modelling study is subject to several limitations that should be considered when interpreting the findings. First, the exclusive use of PubMed as a search database represents a methodological trade-off: while it ensures access to peer-reviewed literature, it may systematically underrepresent research on low-resource settings and countries where publication in internationally indexed journals is less common. Relatedly, this restriction to research published in English, German, and Spanish may lead to the omission of relevant studies published in other languages. This likely disproportionately affects evidence from regions where research is commonly disseminated in local or regional journals. Moreover, stigma associated with mental disorders may contribute to their relative underrepresentation in research, particularly in less industrialized regions. Taken together, the geographic distribution and representativeness of the primary data may be biased towards higher-income regions, and the extent to which these factors influenced the final estimates cannot be fully quantified.

Second, although a broad range of cost components was included to reflect the societal perspective, the resulting estimates should not be interpreted as exhaustive. Certain cost components, such as legal and law enforcement costs, including incarceration costs, and foregone tax revenue, were not analysed, despite potentially representing a substantial burden. For example, a recent report estimated annual fiscal losses from schizophrenia at 56,707 USD per person, of which 39.4% were attributable to criminal justice and homelessness-related costs and 17.5% to lost tax revenue, while health care expenditures accounted for 41.9% of the total costs [48]. An important omission concerns the exclusion of unemployment-related costs. Although unemployment represents a substantial and well-documented consequence of schizophrenia [10], this cost category was excluded to maintain methodological consistency with the broader research framework addressing the global costs of brain disorders. To obtain an even more comprehensive estimate, these components should be incorporated into future research.

Third, this study applies a novel four-step imputation framework to address major data gaps in global cost-of-illness research. By systematically identifying missing data, accounting for country-specific economic contexts, and standardizing cost categories, this framework improves transparency and internal consistency while maintaining cross-country comparability. However, as with all imputation-based approaches, estimates depend on the quality of available primary data and underlying assumptions. More broadly, this limitation reflects challenges inherent to recent macroeconomic modelling approaches applied across multiple medical specialties [37, 49]. Although these models enable global coverage and policy-relevant comparisons, they rely on assumptions, limited data granularity, and an incomplete representation of cross-country heterogeneity. Therefore, these estimates should be interpreted as best-available approximations rather than precise country-level costs. Expanding primary cost-of-illness data, particularly in underrepresented regions, remains essential to reduce uncertainty in future analyses.

Finally, approximately 8% of schizophrenia cases are diagnosed before age 18 [50], meaning that our age threshold of 20 years excludes a proportion of cases whose inclusion would have yielded higher absolute cost estimates. Given that the global annual prevalence of schizophrenia in those aged 19 years or younger is currently estimated at 0.02%, compared to 0.34% across all ages and 0.49% in the 20 + population, the resulting underestimation is likely small to moderate in magnitude [22].

## Conclusion

This study documents an up-to-date, comprehensive assessment of the economic burden of schizophrenia across three income regions globally. The use of a health economic imputation approach to estimate costs for countries lacking primary data expands the existing evidence and fosters cross-regional comparisons. The results reveal notable regional disparities in which schizophrenia is most burdensome in upper- to middle-income countries in economic terms. Direct health care costs account for the largest share of expenditures, with losses from reduced workplace productivity and informal caregiving also playing important roles. The considerable individual disease burden, presenting as high care needs and costs, and productivity losses, emerged as a major driver of the societal burden, resulting in a disproportionately high economic impact given schizophrenia’s relatively small prevalence.

These findings provide policy-makers, researchers, and clinicians with essential evidence to guide future strategies, which may prioritize early prevention, psychosocial interventions, support for informal caregivers, and the development of medical treatments targeting negative symptoms to improve functioning and societal participation among individuals with schizophrenia.

## Supplementary Information

Below is the link to the electronic supplementary material.

Supplementary Material 1

## Data Availability

All data generated or analysed during this study are included in this published article and its supplementary information files.

## Abbreviations

COIN-Eu: Cost of Illness in Neurology in Europe
CPI: Consumer Price Index
DALYs: Disability-Adjusted Life-Years
GBD: Global Burden of Disease
GDP: Gross Domestic Product
GNI: Gross National Income
OECD: Organization for Economic Cooperation and Development
PPP: Purchasing Power Parity
