# Supplementary material for: The global cost of schizophrenia: a health economic modelling study
Source: Int J Ment Health Syst. 2026 Sep 29;20:36. doi: 10.1186/s13033-026-00740-x (PMC13628819; doi:10.1186/s13033-026-00740-x)
Supplement: Supplementary file 1 [file 13033_2026_740_MOESM1_ESM.docx]

Supplementary Materials

[Table 1: Breakdown of Cost Elements	2](#_Toc234016396)

[Table 2: PubMed-based Search Query	2](#_Toc234016397)

[Table 3: Selection Criteria for Economic Sources	3](#_Toc234016398)

[Table 4: Extraction Template	4](#_Toc234016399)

[Figure 1: Materials Used During the Expert Plausibility Check	5](#_Toc234016400)

[Figure 2: PRISMA Flow Chart: Record Selection Process	6](#_Toc234016401)

[Table 5: Number of Extracted Data Points Per Cost Category and Income Group	7](#_Toc234016402)

[Table 6: Selected Economic Sources	8](#_Toc234016403)

[References	19](#_Toc234016404)

[Table 7: Prevalence and Costs of Schizophrenia by Country	24](#_Toc234016405)

[Figure 3: Boxplot Diagrams on Costs Relative to Gross Domestic Product	34](#_Toc234016406)

[Table 8: Parameters Used in Imputation Procedure	35](#_Toc234016407)

[Table 9: Supplementary Analysis to Explore Correlations Between Societal Costs, GDP, and Prevalence	43](#_Toc234016408)

[Predictors of total costs: Multiple linear regression results	50](#_Toc234016409)

# Table 1: Breakdown of Cost Elements

| **Direct costs** | Medical: inpatient care, including emergency department, outpatient care, medications, medical procedures and devices  Non-medical: social services, transportation, and additional costs, such as assisted living |
| --- | --- |
| **Indirect costs** | Costs related to productivity losses: short-term absence from work (sick leave, absenteeism), presenteeism, foregone income, early retirement, premature mortality |
| **Informal care costs** | Assistance in Activities of Daily Living (ADL) and Instrumental Activities of Daily Living (iADL) |

Table detailing included cost sub-components per main cost category.

Studies were included in the economic analysis even when they did not cover all cost subtypes within a given main cost category. However, during the quality check following the initial extraction, and after the expert plausibility check, certain papers were excluded if other studies (from the same country) reporting the same main cost type provided a substantially more comprehensive assessment. This applied to two studies from Germany that reported only costs per hospitalization, one study from Italy that covered only inpatient and drug costs, and one study from Poland and one from Ukraine that reported inpatient care costs exclusively. Additional studies were excluded for reasons unrelated to cost categories — specifically, where non-representative sampling would have introduced substantial selection bias.

In cases where components outside the scope of this research could not be separated from the overarching main cost category (e.g., unemployment cost within indirect costs), a degree of blurriness in the extracted data was tolerated when no—or few—alternatives were available for that country.

# Table 2: PubMed-based Search Query

| **Disease-related search terms** | ("Schizophrenia"[Mesh] OR schizo*[Title]) |
| --- | --- |
| **Economic search terms** | AND ("cost of illness"[MeSH Terms] OR "health expenditures"[MeSH Terms] OR "Health care costs"[MeSH Terms] OR "cost*"[Title] OR "economic*"[Title] OR "financial burden"[Title] OR "expenditure*"[Title] OR "expense*"[Title]) |
| **Publication time filter** | from 2010 - 3000/12/12 |

Table segmenting the search algorithms into disease-related, outcome-related, and publication-related components.

# Table 3: Selection Criteria for Economic Sources

| Inclusion criteria | Exclusion criteria |
| --- | --- |
|  |  |
| Peer-reviewed research | Grey literature, including policy literature, working papers, and government documents |
| Article languages: English, German, Spanish | Published in other languages |
| Study population: adult or general population | Studies reporting data exclusively for minors |
| Study designs: Observational; randomized controlled trials (RCT); cost-effectiveness analysis (CEA); cost-utility analysis (CUA) | Case reports, studies based primarily on modelling, as opposed to primary data acquisition. |
| Studies reporting at least one of the main categories: direct, indirect, and informal care costs | Sources reporting only intangible costs, costs associated with lost tax revenue, costs to caregivers, or costs due to workplace or vehicle-related accidents |
| Scope and granularity of cost assessment: studies reporting data for at least one of the main cost categories (direct, indirect, informal care costs)  Sources reporting diagnosis-related and post-diagnosis costs | Narrow assessments, such as investigations merely focusing on drug costs, were ignored in the presence of a more comprehensive alternative.  Estimates of the total costs without or with unclear breakdown into subcomponents.  Sources reporting only pre-diagnosis costs |
| Prevalence-based cost assessments | Incidence-based cost assessments |
| Study population: patients receiving standard treatment | Samples receiving atypical treatments |
| Costs incurred after disease onset | Costs arising during the premorbid phase |

Table listing the inclusion and exclusion criteria guiding the selection of economic studies during systematic literature review.

# Table 4: Extraction Template

| Country | *Currency* | *Cost year* | *References* | *Remarks 1* | *Remarks 2* | *Quality check complete?* | Direct medical costs | Inpatient Care | Drugs | Outpatient Care | Medical Procedures | Additional costs (medical) |
| --- | --- | --- | --- | --- | --- | --- | --- | --- | --- | --- | --- | --- |
|  |  |  |  |  |  |  |  |  |  |  |  |  |

(continued)

| Direct non-medical costs | Social Services | Adaptations | Transportation | Additional costs (nonmedical) | Long-term care (part of direct costs)* | Total direct costs | Indirect costs | Productivity loss - Or additional indirect costs* | Early retirement | Premature mortality | Informal care | Informal care (Study's definition: Direct = 0; Indirect = 1; category Itself = 2) |
| --- | --- | --- | --- | --- | --- | --- | --- | --- | --- | --- | --- | --- |
|  |  |  |  |  |  |  |  |  |  |  |  |  |

This template was adapted from a previous study on the Costs of Illness in Neurology (COIN).

Column explanations:
"References" documents the citation of each primary source by PMID.
"Remarks 1" was used by the extraction team (LB, LW*, LW, MMM) to communicate requested changes to previous table versions to the researchers responsible for data harmonization and imputation (NM, MA, UW), such as "Informal care costs figure corrected" or "Exclude (non-representative sample, small n)".
"Remarks 2" was initially completed by the researchers conducting the primary data extraction (LB, LW*) and documents key methodological characteristics of each source, including sample size, data acquisition method, and study period, as well as decisions and modifications made during extraction, such as "weighted average between stable and unstable schizophrenia groups was calculated". Further details were added by LW during the quality check.

*including assisted living costs; **including absenteeism and presenteeism costs

# Figure 1: Materials Used During the Expert Plausibility Check

This appraisal template was provided to clinical psychiatry experts together with the model's preliminary results at the following levels of analysis:

- direct (medical and non-medical aggregated), indirect, and informal care costs per patient without imputations [Excel tab **CostsPerPatient_aggr**];
- the same but with direct medical and non-medical separated [**CostsPerPatient**];
- direct (medical and non-medical aggregated), indirect, and informal care costs per patient with imputations [**CostsPerPatient_aggr&imput**];
- direct (medical and non-medical aggregated), indirect, and informal care costs per country with imputations [**CostsPerCountry_aggr&imput**];
- and direct (medical and non-medical aggregated), indirect, and informal care costs per country with imputations, expressed as a percentage of each countries GBD [**%ofGDP_aggr&imput**].

Clinical experts were additionally provided with a document outlining the methodological procedure underlying the economic model, along with the following instructions for completing the appraisal template:

- Are there implausibly high or low total costs in **CostsPerPatient_aggr**? For which countries and in which direction? (appraisal cell 1a)
- Are there implausibly high or low direct, indirect, or informal care costs in **CostsPerPatient_aggr**? For which countries and in which direction? (appraisal cell 1b)
- If 1b applies: → Check only for the countries identified in 1b: Are there implausibly high or low direct medical or direct non-medical costs in **CostsPerPatient**? For which countries and in which direction? (appraisal cell 1c)
- If 1b applies: What effect do the outliers have on the imputed costs at three levels? (appraisal cell 2)
  1. Costs per patient: **CostsPerPatient_aggr&imput**
  2. Costs per country: **CostsPerCountry_aggr&imput**
  3. Costs per country relative to GDP: **%ofGDP_aggr&imput**.
  Possible responses: no/small/medium-sized/large effect on imputed data at level x.
- Remarks: Notes can be added to this cell for each step.

# Figure 2: PRISMA Flow Chart: Record Selection Process

# Table 5: Number of Extracted Data Points Per Cost Category and Income Group

|  | Direct medical costs | Direct nonmedical costs | Total direct costs | Indirect costs | Informal care costs | Income category sum |
| --- | --- | --- | --- | --- | --- | --- |
| Lower income | 0 | 0 | 0 | 0 | 0 | 0 |
| Lower middle income | 1 | 1 | 1 | 2 | 2 | 7 |
| Upper middle income | 12 | 3 | 1 | 3 | 3 | 22 |
| High income | 26 | 8 | 1 | 15 | 6 | 56 |
| Cost category sum | 39 | 12 | 3 | 20 | 11 | 85 |

# Table 6: Selected Economic Sources

| Source | Country | Direct medical costs (DMC) | Direct non-medical costs (DNMC) | Total direct costs  (TDC) | Indirect costs (IC) | Informal care costs (ICC) | Currency | Year | Diagnostic codes | Sample size | Key considerations: |
| --- | --- | --- | --- | --- | --- | --- | --- | --- | --- | --- | --- |
|  |  |  |  |  |  |  |  |  |  |  | *Sources (S); Cost elements (CE) extracted, differentiated by main cost type; Calculation method (C); Disregarded cost elements, i.e. those excluded from further analysis (D); Weighting (W); Sample characteristics (SC); Others (O)* |
| ^1^ | Brazil | 2.528 |  |  |  |  | USD | 2006 |  | 90 | **S:** Medical records.  **CE:** DMC: daily inpatient room rates, first-generation (typical) antipsychotics, second-generation (atypical) antipsychotics.**SC:** Relapsed patients were those who required full-time hospitalization; the mean length of stay was 63 days.  **W:** The average costs were weighted over three hospital systems, hospital perspective (costs of inpatients). |
| ^2^ | Brazil | 1.812 |  |  |  |  | USD PPP | 2000 | ICD-10: F20.0, F20.1, F20.2, F20.3, F20.4, F20.5, F20.6,  F20.8 | 174.31 | **S:** Data were obtained via national administrative databases.  **CE:** DMC: atypical antipsychotics (clozapine, olanzapine, quetiapine, risperidone, ziprasidone), other high-cost drugs, outpatient psychiatric care, other outpatient care, psychiatric hospitalization, other (non-psychiatric) hospitalizations.  **SC:** Adult patients receiving atypical antipsychotics were followed up for 11 years. "Atypical antipsychotics accounted for 79.7% of total costs."  **C:** Inpatient, outpatient, and medication costs were included. The average costs between 2000 and 2010 were calculated because there was no clear index year given and no inflation adjustment but Purchasing power parity. |
| ^3^ | Canada | 19.257 |  |  |  |  | CAD | 2017 | ICD-9:  295.0, 205.5, 295.8, 295.9  ICD-10: F20.0, F20.3, F20.8, F20.9 | 33.176 | **S:** Administrative health data.  **CE:** DMC: emergency department costs, hospitalization costs, physician billing costs, medication costs.  **O:** Only costs of the most recent year of observation were extracted (2017; cross-sectional study). |
| ^4^ | China | 2.863 |  |  |  |  | USD | 2009 | ICD-10:  F20.xx | 2.125 | **S:** Data were obtained via claims databases.  **CE:** DMC: outpatient visits, inpatient admissions, medication costs (antipsychotics and concomitant medications), laboratory tests, diagnostic examinations, medical procedures, nursing care, hospital bed charges.  **C:** CYN costs were converted to USD with an exchange rate from 2009 (6.83 CNY equal to 1 USD) by the authors  **O:** Costs during the first years after diagnosis were included, all-cause costs were calculated, and all direct medical costs subcomponents were considered. |
| ^5^ | China | 51.227 |  |  |  |  | CNY | 2014 | ICD-10:  F20.xx | 2.971 | **S:** Data were obtained via claims databases.  **CE:** DMC: hospitalization costs, outpatient costs, medication costs, diagnostic tests, laboratory examinations, treatment/procedure costs, bed fees, nursing fees, other reimbursed medical services.  **W:** The average costs were weighted over the study period (longitudinal/cohort study).  **O:** Data includes inpatient and outpatient care.  **SC:** The study focused on economically underdeveloped areas of China. |
| ^6^ | China | 9.667 |  |  |  |  | CNY | 2014 | ICD-10:  F20.xx | 39.054 | **CE:** DMC: hospitalization expenses, medications, diagnostic examinations, laboratory tests, treatment/procedure fees, nursing fees, bed charges, rehabilitation fees, other inpatient medical services.  **O:** Only hospitalization costs of the most recent years of observation were extracted (2019; cross-sectional study); 40% of patients paid out of pocket; the average length of stay in 2019 was 38 days. |
| ^7^ | China | 17.593 |  |  |  |  | USD | 2017 |  | 3.296 | **CE:** DMC: outpatient costs and inpatient costs.  **C:** A decision analytic model was calculated to estimate the disease burden of schizophrenia. ¥ were converted to USD according to the currency exchange rate on 31 December 2017 (¥1 = US$0.15) by the authors.  **W:** The average costs were weighted over two treatment groups (injection and oral medication).  **O:** Drug costs were included, but only the two labels, inpatient and outpatient, were given. |
| ^8^ | China | 1.759 |  |  |  |  | USD | 2009 | ICD-10:  F20.xx | 1.131 | **S:** Claims Data were obtained via an insurance database.  **CE:** DMC: Inpatient admissions, outpatient visits, emergency visits, medication costs (typical and atypical antipsychotics and concomitant drugs), laboratory tests, diagnostic examinations, medical procedures, nursing care, hospital bed charges.  **C:** All costs were converted to USD with an exchange rate from 2009 (6.83 CNY to 1 USD) by the authors.  **W:** The average costs were weighted over medication groups (atypical or typical antipsychotics).  **O:** Costs during 1 year after first treatment initiation (including a 90-day washout period) were included, and all direct medical cost subcomponents were considered. |
| ^9^ | China | 715 | 148 |  | 1.010 | 664 | USD | 2010 | DSM-IV (criteria of schizophrenia) | 356 | **S:** Data were obtained with the Economic Burden Questionnaire (EBQ).  **CE:** DMC: Hospitalization costs, outpatient treatment, medication costs, diagnostic examinations, rehabilitation, other medical treatment expenses; DNMC: transportation, accommodation, food expenses during treatment, other patient and family out-of-pocket non-medical expenses; IC: cost due to lost working-days of caregivers; ICC: cost due to lost working-days of caregivers.  **SC:** Sample of 356 individuals from two medical centres. Cases between 16 and 65 years old were included.  **C:** The costs were calculated based on the bottom-up approach; indirect costs were calculated based on the human capital approach.  **D:** costs from damaged properties were not extracted as they are not swithin the scope of this research.  **W:** The average costs were weighted over study groups (rural and urban). |
| ^10^ | Denmark | 21.437 | 375 |  | 41.744 |  | EUR | 2016 | ICD-10:  F20.0 to F20.99 | 12.227 | **S:** Data were obtained from the Danish National Patient Registry.  **CE:** DMC: psychiatric inpatient care, psychiatric outpatient care, somatic inpatient care, somatic outpatient care, primary care services, prescription medications; DMNC: home care by spouses; IC: foregone earnings, early retirement.  **C:** Productivity loss or additional indirect costs were calculated as foregone earnings; early retirement was considered as disability pension in this study.  **D:** Several items were not included in further analysis, for instance, various types of governmental benefits.  **O:** Very comprehensive cost assessments due to national registry, likely contributing to high cost in international comparison. All-cause average costs were calculated over 5 years after diagnosis. |
| ^11^ | France | 7.066 |  |  |  |  | EUR | 2010 | DSM-IV (criteria of schizophrenia) | 288 | **CE:** DMC: psychiatric hospitalizations, general hospitalizations, outpatient psychiatrist visits, psychologist visits, general practitioner visits, specialist physician visits, emergency department visits, medications, laboratory tests, medical procedures.  **SC:** Cases included between 18 and 64 years old.  **C:** The costs were annualized by multiplying by 2.  **D:** The indirect costs were calculated based on the human capital method, an unemployment rate, and an average gross annual pay. There are difficulties in estimating the per-person costs for this category of costs. Therefore, they were excluded. |
| ^12^ | France | 14.995 |  |  |  |  | EUR | 2015 | DSM-IV TR (criteria of schizophrenia) |  | **S:** FondaMental Advanced Centres of Expertise for Schizophrenia cohort.  **CE:** DMC: psychiatric hospitalizations, outpatient consultations, day hospital care, medications, community mental health services, emergency visits. |
| ^13^ | Germany | 5.549 |  |  | 344 |  | EUR | 2006 | ICD-10: F20.xx | 1.449 | **S:** All-cause claims data were obtained from a statutory health insurance's perspective. 2004-2006.  **CE:** DMC: inpatient care, outpatient physician care, outpatient hospital care, rehabilitation, prescription medications, non-physician services, medical aids and devices, sick-pay expenditures; IC: sickness absence.  **C:** Indirect costs are based on sick leave payments. Compared to other methodologies, the results of this approach are low.  **W:** The average costs were weighted between "stable " and "unstable" schizophrenic groups. |
| ^14^ | Germany | 12.471 |  |  | 489 |  | EUR | 2013 | ICD-10-GM: F20.xx | 850 | **S:** Claims data from German Statutory health insurance - BKK funds. Index date, long-acting injectable medication initiation.  **CE:** DMC: psychiatric hospitalizations, outpatient physician visits, long-acting injectable antipsychotics, oral antipsychotics, other prescription medications; IC: sickness benefits.  **C:** Baseline costs were extracted, and the average of two statistical models was created. Indirect costs were considered as sickness benefits; this, compared to other typical approaches, leads to a low estimation. |
| ^15^ | Germany | 12.169 | 582 |  | 3.200 | 7.077 | EUR | 2008 | ICD-10-GM: F20.xx | 8.224 | **S:** Data were obtained via a sickness fund claims database.  SC: Individuals with at least one inpatient diagnosis between 2005 and 2008.  **CE:** DMC: Inpatient care, outpatient care, physician visits, hospital services, prescription medications, rehabilitation, therapeutic aids; DNMC: administration, investments, travel expenses, other; IC: sick leave, disability benefits, productivity loss due to work incapacity, premature retirement; ICC: Informal family care.  **C:** The costs are reported from the payer's perspective and societal perspective (preferred). The indirect costs are based on the friction cost method. Informal care costs were included in this study as direct non-medical costs, but recategorized by us. |
| ^16^ | Hungary |  |  |  | 9.912 |  | EUR | 2018 |  | 78 | **C:** The costs were calculated based on the human capital approach, modelling via multiple linear regression.  **CE:** IC: absenteeism, presenteeism, disability pension, early retirement, productivity losses.  **O:** This study analyses data on 11 chronic illnesses, including schizophrenia |
| ^17^ | India |  |  | 10.971 | 15.734 | 10.919 | Indian Rupees | 2010 | DSM-IV (criteria of schizophrenia) | 53 | **S:** Data were obtained from a large multispecialty tertiary-care hospital via a detailed semi-structured interview.  **CE:** TDC: outpatient consultations, medications, laboratory investigations; transportation, food expenses, accommodation; IC: loss of wages of patients, loss of wages of caregivers; ICC: caregiver time spent accompanying patients to treatment.  **S:** Sample size, n=53. Setting: Outpatients of a psychiatric hospital.  **O:** direct costs were predominantly OOP expenses, as typical for India [extracted from Table 4 in paper], indirect costs were calculated based on the human capital approach as loss of income of the patient [extracted from Table 5 in paper], informal care costs were calculated based on the human capital approach as loss of income of the caregiver [extracted from Table 5 in paper], indirect and informal care costs were annualized as such: /9 (9 months study period) *12.  **O:** This study also has data on bipolar disorders. |
| ^18^ | Indonesia |  |  | 4.899.660 |  |  | IDR | 2018 | ICD-10: F20.xx | 137 | **CE:** TDC: antipsychotic medications, concomitant medications, outpatient consultation fees, dispensing fee.  **SC:** Only new outpatients who had only received drugs for treatment were included; patients who had (also) received psychotherapy and had comorbid mental diseases were excluded.  **C:** Direct costs were drug, administrative, and doctor consultation fees [extracted from Table 6]. The average annual cost per year was preferred when extracting.  **O:** Cross-sectional study (therefore extracted only costs of most recent year). |
| ^19^ | Italy | 6.446 |  |  | 5.853 | 723 | EUR | 2014 | ICD-10: F20.xx | 303.913 | **CE:** DMC: hospitalizations, residential and semi-residential psychiatric care, outpatient services, medications, diagnostic tests; IC: disability, premature mortality; ICC: Informal caregiving time provided by family members.  **C:** A probabilistic cost of illness model was calculated, fractions of total costs were calculated based on the percentages reported in paper, per patient costs calculated by dividing by estimated prevalence reported in the paper, direct medical costs include hospitalizations, drugs and outpatient costs, indirect costs include loss of productivity and disability pension - fraction of lost productivity to caregiver (* 0.89 = -11%). |
|  |  |  |  |  |  |  |  |  |  |  |  |
| ^20^ | Italy |  |  | 29.138 | 11.415 |  | EUR | 2018 |  | 523 | **S:** Data were obtained from the Department of Mental Health of Bari.  **CE:** TDC: psychiatric visits, psychological interventions, nursing care, hospitalizations, day hospital services, residential care, medications, laboratory tests, diagnostic examinations, rehabilitation services, patient transportation; IC: productivity loss due to inability to work; ICC: informal caregiving time.  **SC:** Subjects were aged between 18 and 65 years.  **C:** Direct costs (medical and non-medical) were assessed comprehensively, likely prevalence-based.  **D:** Due to the composition of the direct non-medical costs, it was preferred to extract the total direct costs directly. |
| ^21^ | Italy | 4.157 |  |  |  |  | EUR | 2010 | ICD-10: F20.xx  F20.xx  ICD-9:  295.xx | 7.457 | **S:** Data were obtained from administrative databases of 16 Local Health Units, resulting in a multicentre study.  **CE:** DMC: antipsychotic medications, concomitant medications, hospitalizations, outpatient specialist visits, diagnostic services.  **SC:** Subjects were aged 18 years and older  **O:** Not disease-specific and not all-cause costs, but disease-related costs were reported. |
| ^22^ | Japan |  |  |  | 1.813.921 |  | JPY | 2019 |  | 178 | **CE:** IC: absenteeism and presenteeism.  **SC:** Subjects were aged 18 years or older.  **C:** The human capital approach was implemented to estimate the indirect costs. The total work productivity impairment consisted of absenteeism and presenteeism.  **W:** The average costs were weighted over comorbidity groups.  **O:** Unadjusted values were extracted (Table 3). |
| ^23^ | Japan | 977.736 | 4.435 |  | 2.556.580 |  | JPY | 2008 | ICD-10:  F20.0 to F20.9 |  | **S:** Data were obtained from Patient Survey, the Survey of Medical Care Activities in Public Health Insurance.  **CE:** DMC: Inpatient and outpatient health care costs under health insurance, incl. prescription medications and psychiatric services; DNMC: social service costs; IC: morbidity-related productivity loss, mortality-related productivity loss.  **C:** The costs are reported as a population total, divided by prevalence reported in the paper (404000+380000) to obtain per-patient costs.  Indirect costs include morbidity (presenteeism, absenteeism, and unemployment costs) and mortality (expected lifetime earnings loss) costs. |
| ^24^ | Malaysia | 7.349 | 463 |  | 14.989 | 5.542 | MYR | 2015 | DSM-5 (criteria of schizophrenia) |  | **S:** Data were obtained via medical chart review from one hospital (micro-costing technique).  **CE:** DMC: hospitalization, outpatient visits, medications, community mental health services; DNMC: transportation; IC: productivity loss due to unemployment, absenteeism, premature mortality; ICC: informal caregiving time.  **C:** The cost per-patient per year was calculated by dividing by the reported number of schizophrenia patients. |
| ^25^ | Mexico | 44.263 |  |  |  |  | MXN | 2017 |  | 306 | **CE:** DMC: outpatient physician consultations, medications, laboratory tests.  **SC:** Patients with schizophrenia (schizoaffective disorders) in ambulatory care attended Family Medicine. 18 years old and over.  **C:** Costs include family medicine consultations, inter-consultations, medications, medical supplies, and the time of use of installed capacity for one year.  **O:** The paper is written in Spanish. |
| ^26^ | New Zealand | 16.695 | 14.760 |  |  |  | NZD | 2015 | DSM-IV:  295.1 to 295.9, excluding 295.7  ICD-10-AM:  F20.0 to F20.9  ICD-9:  295  to 295.9, excluding 295.7 | 18.096 | **CE:** DMC: hospital admissions, emergency department (ED) attendances, outpatient visits, community pharmaceuticals, laboratory tests, general medical subsidies, specialty mental health services; DNMC: social welfare benefits, social housing.  S: Data were obtained from the Integrated Data Infrastructure  **CE:** DMC:  **O:** All-cause data, which can be interpreted as prevalence-based data, were extracted from Appendix Table 2. Direct non-medical costs include any social welfare benefit and social housing. |
| ^27^ | Nigeria | 350 | 120 |  | 225 | 118 | USD | 2011 |  | 100 | **S:** Data were obtained from one teaching hospital.  **CE:** DMC: consultation fees, medications, laboratory investigations, hospital admissions; DNM: transportation, feeding, accommodation; IC: loss of patient income, loss of caregiver income; ICC: caregiver time spent accompanying and caring for patients.  **C:** Direct medical costs include costs for religious, traditional, and other alternative treatments (18.1%), criminal justice costs [not presented in this table] include use of law enforcement agents (Table 3). |
| ^28^ | Norway | 400.487 | 120.353 |  | 77.613 |  | NOK | 2012 | ICD-10:  F20.xx, F25.xx | 8.399 | **S:** Data were obtained from comprehensive and mandatory health and welfare registers in Norway.  **CE:** DMC: mental healthcare services, somatic healthcare services, prescription medications; DNMC: community care services; IC: employment loss, disability pension, productivity loss.  **C:** Total costs were divided by the prevalence reported in the paper to obtain the costs per patient.  Direct medical costs include mental health treatment, direct non-medical costs include community care services (including home nursing/assistance), and indirect costs include lost productivity and social security. Costs resulting from premature mortality and presenteeism are not included; only absenteeism and disability pension are considered, which leads to a conservative estimate.  **D:** The unemployment costs and lost taxes were excluded from our calculations to align with our approach. |
| ^29^ | South Korea | 4.293.128 | 1.186.483 |  | 6.705.226 | 3.279.399 | KRW | 2016 |  | 202.345 | **S:** Data were obtained from HI claims, surveys, and administrative sources. Data were extracted for 2016 only.  **CE:** DMC: Inpatient care, outpatient care, medications; DNMC: transportation; IC: productivity loss due to morbidity, productivity loss due to premature mortality; ICC: informal caregiving time.  **C:** Costs were only given on a national annual level. Therefore, per-person costs were calculated by dividing them with the treated prevalence of schizophrenia in South Korea in 2016, as shown in the study. Direct medical costs include OOP costs (uncovered), direct non-medical costs include rehabilitation/sanatoria (which we consider a medical cost category), and indirect costs were calculated based on the human capital approach, unemployment, reduced productivity, premature mortality, and criminal justice costs, including incarceration.  **D:** The unemployment costs were excluded to align with our approach. |
| ^30^ | Sweden | 143.169 |  |  |  |  | SEK | 2006 |  | 164 | **S:** Data were obtained from a practice chart review, a multicentre study (from 9 sites). This publication assesses the implications of switching to long-acting injectables  **CE:** DMC: long-acting risperidone injections, psychiatric hospitalizations, outpatient visits.  **SC:** The study sample included patients with schizophrenia or schizoaffective disorder  **W:** A weighted average between before and after switch groups was calculated.  **O:** Data are for schizophrenia and schizoaffective disorder combined.  This cost-effectiveness analysis, CEA study on the costs of switching to long-acting injection antipsychotics includes only inpatient and medication costs, costs can be considered as disease-related (but not exclusively disease-specific). |
| ^31^ | Sweden | 9.728 | 12.400 |  | 33.020 |  | EUR | 2008 | ICD-10:  F20.xx, F21.xx, F23.1, F23.2, F23.8, F23.9, F25.1, F25.8, F25.9 | 2.161 | **S:** Hospital-based and national registry data were obtained. 2006-2008.  **CE:** DMC: inpatient care, outpatient care, prescription medications, community psychiatric services; DNMC: municipal housing services, supported accommodation, social care services; IC: productivity loss due to sick leave, disability pension, premature mortality; ICC: informal caregiving time.  **C:** The paper states that the costs are based on sick leaves and early retirement costs. Still, the calculation process is not straightforward, particularly considering that the result is high compared to other studies. A disaggregation of the costs is impossible. |
| ^32^ | Switzerland | 9.507 | 3.464 |  | 25.108 | 1.329 | EUR | 2012 | ICD-10:  F20.xx, F23.xx, F25.xx |  | **S:** Data were obtained from hospital registry, an outpatient physician survey, and health insurance claims.  **CE:** DMC: inpatient care, outpatient care, physician visits, medications, psychiatric services; DNMC: residential care, sheltered housing, transportation; IC: productivity loss, disability, premature mortality; ICC: Informal caregiving time.  **C:** A prevalence-based bottom-up approach was used. Informal care costs were considered in the publication as direct non-medical costs but moved by us to the informal care cost category.  Indirect costs include presenteeism and social security contributions by employers. |
| ^33^ | Taiwan (Province of China) | 125.891 |  |  |  |  | NTD | 2008 | ICD-9-CM:  295.xx | 16.805 | **S:** Data were obtained from the National Health Insurance Research Database.  **CE:** DMC: inpatient care, outpatient care, emergency department visits.  **C:** The costs from the 36-month (3-year) observation period were divided by 3 to annualize them.  **W:** The average costs were weighted over the two age groups (younger than 65 and older than 65 years). |
| ^34^ | Taiwan (Province of China) | 4.110 |  |  |  |  | USD PPP | 2010 | ICD-9-CM:  295.xx | 34.658 | **S:** Reimbursement data were obtained from the National Health Insurance Research Database.  **CE:** DMC: lifetime inpatient care, outpatient care, healthcare expenditures.  **C:** Modelling, simulation, and extrapolation were used to estimate the costs.  **W:** The average costs were weighted over different age groups and sexes (Table 2)  **O:** Costs are reported per life year and not per year lived with the disease (prevalence-based costs); therefore, the estimation is rather conservative. |
| ^35^ | Thailand | 426 |  |  |  |  | USD | 2014 | ICD-10:  F20.xx | 582 | **S:** Data were obtained from one university hospital in Bangkok.  **CE:** DMC: hospitalizations, outpatient visits, medications.  **SC:** The subjects were aged 18–65 years.  **C:** The focus of the publication is on the effect of medication adherence on medical costs.  W: The average costs were weighted over all adherence groups.  **O:** The study focused on a hospital perspective. Since only all-cause direct medical costs are included, the study investigates the effect of medication adherence on medical costs. |
| ^36^ | Thailand | 21.700 | 1.700 |  | 53.000 | 11.000 | THB | 2008 | ICD-10:  F20.xx, F25.xx | 307 | **S:** Data were obtained via a survey among people in treatment for schizophrenia; data were extracted from Table 4 in the paper.  **CE:** DMC: hospitalizations, outpatient care, medications, psychiatric services; DNMC: transportation; IC: productivity loss due to unemployment, absenteeism, premature mortality; ICC: informal caregiving time.  **O:** Direct non-medical costs include travel costs. |
| ^37^ | United States of America | 23.349 |  |  |  |  | USD | 2011 | ICD-9:  295.xx | 18.136 | **S:** Health claims data were obtained from Medicaid databases from 5 states.  **CE:** DMC: atypical antipsychotic medications, inpatient admissions, emergency department visits, outpatient visits.  **SC:** The study sample included patients with at least two diagnoses for schizophrenia.  **W:** The average costs were weighted over relapse and non-relapse groups.  **O:** The study included all-cause costs, the baseline period was defined as 1 year before the first diagnosis of schizophrenia (index date), and the study contains a comprehensive assessment of subcategories of the direct medical costs. |
| ^38^ | United States of America | 21.672 |  |  |  |  | USD | 2011 | ICD-9:  295.xx | 8.985 | **S:** Claims data were obtained from the Truven Health Market Scan database, and the all-cause costs were extracted from the snapshot analysis of patients with newly diagnosed and chronic schizophrenia  **CE:** DMC: inpatient admissions, outpatient visits, emergency department visits, physician services, prescription medications.  **C:** The costs were multiplied by 12 to annualize. |
| ^39^ | United States of America | 32.920 |  |  |  |  | USD | 2020 | ICD-10-CM:  F20.xx, F21.xx, F25.xx | 688.437 | **S:** Data were obtained via Transformed Medicaid Statistical Information System (T-MSIS) Analytic Files (TAF) for 45 of 50 states.  **CE:** DMC: inpatient care, outpatient care, emergency department visits, physician services, pharmacy claims.  **SC:** The study sample included adult Medicaid beneficiaries with at least two diagnoses of schizophrenia.  **O:** All-cause costs and prevalence-based data were considered. |
| ^40^ | United States of America | 16.752 |  |  |  |  | USD | 2007 | ICD-9:  295.xx | 3.966 | **S:** Claims data were obtained via the PharMetrics Integrated Database for the period 1998–2007.  **CE:** DMC: hospitalizations, outpatient visits, emergency department visits, medications.  **W:** The average costs were weighted over recently diagnosed and chronic patients.  **C:** Only costs from the retrospective year were extracted (Figure 2), therefore approximating the average prevalent patient.  **O:** For chronic patients, there was no significant difference between costs in the year preceding the index event and the year after the index event (both years with diagnosis). |
| ^41^ | United States of America |  |  |  | 1.664 | 15.095 | USD | 2013 | ICD-9:  295.xx |  | **S:** Data were obtained from Truven Health Analytics MarketScan Commercial Claims and Encounters, Medicare Supplemental, and Medicaid Multistate databases.  **CE:** DMC: inpatient care, outpatient care, emergency department visits, physician services, prescription medications, long-term care; DNMC: homeless services, supported housing, law enforcement, incarceration; IC: reduced workplace productivity, absenteeism, premature mortality; ICC: informal caregiving time.  **C:** Costs reported on country-level were divided by the prevalence reported in the paper (3 477 417) to calculate per-patient per year costs.  **D:** All these data represent excess costs compared to the matched control group; therefore, the direct medical and non-medical costs were excluded (because they represent the excess costs), and only the remaining cost types were considered.  The unemployment costs were removed from the indirect costs to align with our approach.  **O:** Direct non-medical costs include homeless shelters, research and training costs were not extracted (excluded), direct medical costs include long-term care (only 0.4% of total costs), indirect costs include unemployment, productivity loss and premature mortality (suicide),  data were extracted from Table 1 |
| ^42^ | United States of America |  |  |  | 6.958 | 28.761 | USD | 2019 | ICD-10-CM:  F20.xx, F25.xx |  | **S:** Data were obtained from IBM Watson Health MarketScan databases.  **CE:** IC: absenteeism, presenteeism, premature mortality; ICC: informal caregiving time.  **C:** 2019 prevalence data from the article was used to calculate the per-patient per year figure.  **D:** The study also reported direct medical and non-medical costs. Direct medical costs were not extracted as they included long-term care (considered a non-medical cost here) and are based on excess costs; the difference between patients with schizophrenia and matched controls (the approach employed here favoured an all-cause assessment). Direct non-medical costs were also excluded because they include research and training costs, criminal justice costs such as incarceration, legal, judicial services and police protection, which were not in the scope of the study's aim. The unemployment costs were removed from the indirect costs to align with our approach.  **O:** Indirect costs were based on the human capital approach and included the value of quality-adjusted life-years lost (which we categorize as intangible), unemployment, productivity loss and premature mortality. |
| ^43^ | United States of America | 25.323 |  |  |  |  | USD | 2005 | ICD-9-CM:  295.xx | 5.694 | **S:** Data for this study on long-term injectable antipsychotics were obtained from the Thomson Reuters MarketScan® Research Medicaid database.  **CE:** DMC: long-acting injectable antipsychotics, inpatient admissions, outpatient visits, emergency department visits, pharmacy costs.  **SC:** The study sample included patients aged 13 years and older.  **C:** The 6-month baseline costs (before LAI initiation) were multiplied by 2 to annualize the results. |
| ^44^ | United States of America | 16.035 |  |  |  |  | USD | 2000 |  | 1.557 | **S:** Data were obtained from a prospective, observational, non-interventional study of schizophrenia in the United States (US-SCAP) between 7/1997 and 9/2003. Participants were recruited from diverse geographic areas. The six participating regional sites represented large systems of care and usual care settings  **CE:** DMC: psychiatric hospitalizations, outpatient visits, emergency department visits, medications.  **SC:** The study sample included patients with schizophrenia, schizoaffective, or schizophreniform disorder (= broader than just schizophrenia population).  **W:** The average costs were weighted over relapsers and non-relapsers. |
| ^45^ | United States of America | 28.644 |  |  |  |  | USD | 2018 | ICD-9-CM:  295.xx  ICD-10-CM:  F20.xx, F21.xx, F25.xx | 158.763 | **S:** Data were obtained from Medicare Supplemental and Medicaid claims databases.  **CE:** DMC: inpatient care, outpatient care, emergency department visits, physician services, pharmacy claims.  **SC:** The analysis was made across six states. The study sample included patients with at least two claims on different days with a diagnosis of schizophrenia.  **C:** The study reported costs 12 months after the index date (Figure 4). The index date was the most recent eligible claim with a diagnosis of schizophrenia. |
| ^46^ | United States of America | 18.995 |  |  |  |  | USD | 2008 | ICD-9-CM:  295.xx | 147 | **S:** Data was obtained from Homson Medstat MarketScan commercial claims and encounters databases. Cost-effectiveness analyses of depot antipsychotics.  **CE:** DMC: depot antipsychotics, inpatient hospitalizations, outpatient visits, emergency department visits, healthcare expenditures.  **C:** The added costs during the period of 6 months pre- to 6 months post-initiation on any depot antipsychotic were considered.  **D:** It is unclear if costs are per patient or for the whole sample of 147 persons. Only total costs were given. Therefore, the costs were divided by the number of patients included in the study (147). |

## References

1.	Daltio CS, Mari JJ, Ferraz MB. Direct medical costs associated with schizophrenia relapses in health care services in the city of São Paulo. *Rev Saude Publica*. 2011;45(1):14-23. doi:10.1590/s0034-89102010005000049

2.	Barbosa WB, Costa J de O, de Lemos LLP, et al. Costs in the Treatment of Schizophrenia in Adults Receiving Atypical Antipsychotics: An 11-Year Cohort in Brazil. *Appl Health Econ Health Policy*. 2018;16(5):697-709. doi:10.1007/s40258-018-0408-4

3.	Stewart AJ, Patten SB, Fiest KM, Williamson TS, Wick JP, Ronksley PE. 10-Year Trends in Healthcare Spending among Patients with Schizophrenia in Alberta, Canada. *Can J Psychiatry Rev Can Psychiatr*. 2022;67(9):723-733. doi:10.1177/07067437221082885

4.	Wu J, He X, Liu L, et al. Health care resource use and direct medical costs for patients with schizophrenia in Tianjin, People’s Republic of China. *Neuropsychiatr Dis Treat*. 2015;11:983-990. doi:10.2147/NDT.S76231

5.	Zhang H, Sun Y, Zhang D, Zhang C, Chen G. Direct medical costs for patients with schizophrenia: a 4-year cohort study from health insurance claims data in Guangzhou city, Southern China. *Int J Ment Health Syst*. 2018;12:72. doi:10.1186/s13033-018-0251-x

6.	Li J, Du H, Dou F, et al. A study on the changing trend and influencing factors of hospitalization costs of schizophrenia in economically underdeveloped areas of China. *Schizophrenia*. 2023;9(1):4. doi:10.1038/s41537-023-00331-6

7.	Zhong Q, Tan Y, Chen W, et al. Disease burden of schizophrenia patients visiting a Chinese regional mental health centre. *J Comp Eff Res*. 2020;9(7):469-481. doi:10.2217/cer-2019-0129

8.	He X, Wu J, Jiang Y, et al. Health care resource utilization and direct medical costs for patients with schizophrenia initiating treatment with atypical versus typical antipsychotics in Tianjin, China. *BMC Health Serv Res*. 2015;15:149. doi:10.1186/s12913-015-0819-y

9.	Zhai J, Guo X, Chen M, Zhao J, Su Z. An investigation of economic costs of schizophrenia in two areas of China. *Int J Ment Health Syst*. 2013;7(1):26. doi:10.1186/1752-4458-7-26

10.	Hastrup LH, Simonsen E, Ibsen R, Kjellberg J, Jennum P. Societal Costs of Schizophrenia in Denmark: A Nationwide Matched Controlled Study of Patients and Spouses Before and After Initial Diagnosis. *Schizophr Bull*. 2020;46(1):68-77. doi:10.1093/schbul/sbz041

11.	Sarlon E, Heider D, Millier A, et al. A prospective study of health care resource utilisation and selected costs of schizophrenia in France. *BMC Health Serv Res*. 2012;12:269-276. doi:10.1186/1472-6963-12-269

12.	Laidi C, Prigent A, Plas A, et al. Factors associated with direct health care costs in schizophrenia: Results from the FACE-SZ French dataset. *Eur Neuropsychopharmacol J Eur Coll Neuropsychopharmacol*. 2018;28(1):24-36. doi:10.1016/j.euroneuro.2017.11.020

13.	Zeidler J, Slawik L, Fleischmann J, Greiner W. The costs of schizophrenia and predictors of hospitalisation from the statutory health insurance perspective. *Health Econ Rev*. 2012;2(1):9. doi:10.1186/2191-1991-2-9

14.	Mahlich J, Olbrich K, Wilk A, Wimmer A, Wolff-Menzler C. Hospitalization Rates and Therapy Costs of German Schizophrenia Patients Who are Initiated on Long-Acting Injectable Medication: A Mirror-Image Study. *Clin Drug Investig*. 2020;40(4):355-375. doi:10.1007/s40261-020-00900-y

15.	Frey S. The economic burden of schizophrenia in Germany: a population-based retrospective cohort study using genetic matching. *Eur Psychiatry J Assoc Eur Psychiatr*. 2014;29(8):479-489. doi:10.1016/j.eurpsy.2014.04.003

16.	Rashdan O, Brodszky V. Productivity Loss in Patients With Chronic Diseases: A Pooled Economic Analysis of Hungarian Cost-of-Illness Studies. *Value Health Reg Issues*. 2020;22:75-82. doi:10.1016/j.vhri.2020.07.572

17.	Somaiya M, Grover S, Chakrabarti S, Avasthi A. Comparative study of cost of care of outpatients with bipolar disorder and schizophrenia. *Asian J Psychiatry*. 2014;12:125-133. doi:10.1016/j.ajp.2014.08.003

18.	Puspitasari IM, Sinuraya RK, Rahayu C, et al. Medication Profile and Treatment Cost Estimation Among Outpatients with Schizophrenia, Bipolar Disorder, Depression, and Anxiety Disorders in Indonesia. *Neuropsychiatr Dis Treat*. 2020;16:815-828. doi:10.2147/NDT.S240058

19.	Marcellusi A, Fabiano G, Viti R, et al. Economic burden of schizophrenia in Italy: a probabilistic cost of illness analysis. *BMJ Open*. 2018;8(2):e018359. doi:10.1136/bmjopen-2017-018359

20.	Latorre V, Messeni Petruzzelli A, Uva AE, Ranaudo C, Semisa D. Unveiling the actual cost of Schizophrenia: An Activity-Based Costing (ABC) approach. *Int J Health Plann Manage*. 2022;37(3):1366-1380. doi:10.1002/hpm.3405

21.	Degli Esposti L, Sangiorgi D, Mencacci C, et al. Pharmaco-utilisation and related costs of drugs used to treat schizophrenia and bipolar disorder in Italy: the IBIS study. *BMC Psychiatry*. 2014;14:282. doi:10.1186/s12888-014-0282-z

22.	Baba K, Guo W, Chen Y, Nosaka T, Kato T. Burden of schizophrenia among Japanese patients: a cross-sectional National Health and Wellness Survey. *BMC Psychiatry*. 2022;22(1):410. doi:10.1186/s12888-022-04044-5

23.	Sado M, Inagaki A, Koreki A, et al. The cost of schizophrenia in Japan. *Neuropsychiatr Dis Treat*. 2013;9:787-798. doi:10.2147/NDT.S41632

24.	Teoh SL, Chong HY, Abdul Aziz S, et al. The economic burden of schizophrenia in Malaysia. *Neuropsychiatr Dis Treat*. 2017;13:1979-1987. doi:10.2147/NDT.S137140

25.	González-Pérez B, Salas-Flores R, de León-Escobedo R, Carrillo-Aguiar LA, Salas-Galarza A. [Cost to ambulatory care for schizophrenia in Family Medicine]. *Rev Medica Inst Mex Seguro Soc*. 2019;57(4):213-217.

26.	Gibb S, Brewer N, Bowden N. Social impacts and costs of schizophrenia: a national cohort study using New Zealand linked administrative data. *N Z Med J*. 2021;134(1537):66-83.

27.	Oloniniyi IO, Akinsulore A, Aloba OO, Mapayi BM, Oginni OA, Makanjuola R. Economic Cost of Schizophrenia in a Nigerian Teaching Hospital. *J Neurosci Rural Pract*. 2019;10(1):39-47. doi:10.4103/jnrp.jnrp_210_18

28.	Evensen S, Wisløff T, Lystad JU, Bull H, Ueland T, Falkum E. Prevalence, Employment Rate, and Cost of Schizophrenia in a High-Income Welfare Society: A Population-Based Study Using Comprehensive Health and Welfare Registers. *Schizophr Bull*. 2016;42(2):476-483. doi:10.1093/schbul/sbv141

29.	Jo M, Kim HJ, Rim SJ, Lee MG, Kim CE, Park S. The cost-of-illness trend of schizophrenia in South Korea from 2006 to 2016. *PloS One*. 2020;15(7):e0235736. doi:10.1371/journal.pone.0235736

30.	Willis M, Svensson M, Löthgren M, Eriksson B, Berntsson A, Persson U. The impact on schizophrenia-related hospital utilization and costs of switching to long-acting risperidone injections in Sweden. *Eur J Health Econ HEPAC Health Econ Prev Care*. 2010;11(6):585-594. doi:10.1007/s10198-009-0215-9

31.	Ekman M, Granstrom O, Omerov S, Jacob J, Landen M. The societal cost of schizophrenia in Sweden. *J Ment Health Policy Econ*. 2013;16(1):13-25.

32.	Pletscher M, Mattli R, von Wyl A, Reich O, Wieser S. The Societal Costs of Schizophrenia in Switzerland. *J Ment Health Policy Econ*. 2015;18(2):93-103.

33.	Pan YJ, Kuo KH, Yeh LL. Healthcare cost, service use and mortality in major psychiatric disorders in Taiwan. *J Affect Disord*. 2019;246:112-120. doi:10.1016/j.jad.2018.12.046

34.	Lêng CH, Chou MH, Lin SH, Yang YK, Wang JD. Estimation of life expectancy, loss-of-life expectancy, and lifetime healthcare expenditures for schizophrenia in Taiwan. *Schizophr Res*. 2016;171(1-3):97-102. doi:10.1016/j.schres.2016.01.033

35.	Dilokthornsakul P, Thoopputra T, Patanaprateep O, Kongsakon R, Chaiyakunapruk N. Effects of medication adherence on hospitalizations and healthcare costs in patients with schizophrenia in Thailand. *SAGE Open Med*. 2016;4:2050312116637026. doi:10.1177/2050312116637026

36.	Phanthunane P, Whiteford H, Vos T, Bertram M. Economic burden of schizophrenia: empirical analyses from a survey in Thailand. *J Ment Health Policy Econ*. 2012;15(1):25-32.

37.	Lafeuille MH, Gravel J, Lefebvre P, et al. Patterns of relapse and associated cost burden in schizophrenia patients receiving atypical antipsychotics. *J Med Econ*. 2013;16(11):1290-1299. doi:10.3111/13696998.2013.841705

38.	Fitch K, Iwasaki K, Villa KF. Resource utilization and cost in a commercially insured population with schizophrenia. *Am Health Drug Benefits*. 2014;7(1):18-26.

39.	Patel C, Pilon D, Gupta D, et al. National and regional description of healthcare measures among adult Medicaid beneficiaries with schizophrenia within the United States. *J Med Econ*. 2022;25(1):792-807. doi:10.1080/13696998.2022.2084234

40.	Nicholl D, Akhras KS, Diels J, Schadrack J. Burden of schizophrenia in recently diagnosed patients: healthcare utilisation and cost perspective. *Curr Med Res Opin*. 2010;26(4):943-955. doi:10.1185/03007991003658956

41.	Cloutier M, Aigbogun MS, Guerin A, et al. The Economic Burden of Schizophrenia in the United States in 2013. *J Clin Psychiatry*. 2016;77(6):764-771. doi:10.4088/JCP.15m10278

42.	Kadakia A, Catillon M, Fan Q, et al. The Economic Burden of Schizophrenia in the United States. *J Clin Psychiatry*. 2022;83(6):22m14458. doi:10.4088/JCP.22m14458

43.	Bera R, Offord S, Zubek D, et al. Impact on healthcare resource usage and costs among Medicaid-insured schizophrenia patients after initiation of treatment with long-acting injectable antipsychotics. *J Med Econ*. 2013;16(4):522-528. doi:10.3111/13696998.2013.771641

44.	Ascher-Svanum H, Zhu B, Faries DE, et al. The cost of relapse and the predictors of relapse in the treatment of schizophrenia. *BMC Psychiatry*. 2010;10:2. doi:10.1186/1471-244X-10-2

45.	Pilon D, Patel C, Lafeuille MH, et al. Prevalence, incidence and economic burden of schizophrenia among Medicaid beneficiaries. *Curr Med Res Opin*. 2021;37(10):1811-1819. doi:10.1080/03007995.2021.1954894

46.	Peng X, Ascher-Svanum H, Faries D, Conley RR, Schuh KJ. Decline in hospitalization risk and health care cost after initiation of depot antipsychotics in the treatment of schizophrenia. *Clin Outcomes Res CEOR*. 2011;3:9-14. doi:10.2147/CEOR.S16061

# Table 7: Prevalence and Costs of Schizophrenia by Country

| Country (with imputed values) | GNI group | Adult population (20+ years) | Prevalence per 100,000 adults | of that % working age (20-64 years) | Per-patient direct costs (a) | Per-patient informal care costs (b) | Per-patient indirect costs (c) | Per-patient total costs | Direct costs per country (a) (million $) | Informal care costs per country (b) (million $) | Indirect costs per country (c) (million $) | Total costs per country (million $) | % GBD |
| --- | --- | --- | --- | --- | --- | --- | --- | --- | --- | --- | --- | --- | --- |
| Andorra(a,b,c) | H | 68,211 | 436 | 90 | 8,651 | 2,116 | 14,778 | 25,546 | 3 | 1 | 4 | 7 | 0.23 |
| Antigua and Barbuda(a,b,c) | H | 64,698 | 367 | 92 | 5,727 | 1,401 | 6,551 | 13,679 | 1 | 0 | 1 | 3 | 0.19 |
| Australia(a,b,c) | H | 18,829,490 | 571 | 89 | 12,775 | 3,125 | 19,646 | 35,546 | 1,373 | 336 | 1,880 | 3,588 | 0.26 |
| Austria(a,b,c) | H | 7,161,958 | 404 | 86 | 13,450 | 3,290 | 17,905 | 34,644 | 390 | 95 | 448 | 932 | 0.21 |
| Bahamas(a,b,c) | H | 265,686 | 365 | 93 | 7,412 | 1,813 | 11,661 | 20,886 | 7 | 2 | 11 | 20 | 0.15 |
| Bahrain(a,b,c) | H | 1,085,845 | 447 | 98 | 5,171 | 1,265 | 9,251 | 15,686 | 25 | 6 | 44 | 75 | 0.19 |
| Barbados(a,b,c) | H | 228,196 | 393 | 88 | 8,121 | 1,986 | 6,795 | 16,902 | 7 | 2 | 5 | 14 | 0.27 |
| Belgium(a,b,c) | H | 8,865,390 | 398 | 86 | 13,736 | 3,360 | 16,677 | 33,773 | 485 | 119 | 504 | 1,107 | 0.21 |
| Bermuda(c) | H | 52,135 | 371 | 86 |  |  | 41,535 | 41,535 |  |  | 7 | 7 | 0.09 |
| Brunei Darussalam(a,b,c) | H | 310,543 | 428 | 96 | 2,785 | 681 | 10,995 | 14,461 | 4 | 1 | 14 | 19 | 0.14 |
| Canada(b,c) | H | 13,861,097 | 359 | 87 | 16,103 | 3,419 | 16,567 | 36,089 | 1,974 | 419 | 1,772 | 4,165 | 0.24 |
| Chile(a,b,c) | H | 6,304,396 | 352 | 92 | 12,030 | 2,943 | 5,232 | 20,205 | 703 | 172 | 281 | 1,156 | 0.42 |
| Croatia(a,b,c) | H | 3,428,367 | 330 | 86 | 8,996 | 2,200 | 5,483 | 16,678 | 102 | 25 | 53 | 180 | 0.29 |
| Cyprus(a,b,c) | H | 1,040,887 | 416 | 89 | 9,035 | 2,210 | 10,519 | 21,764 | 39 | 10 | 41 | 89 | 0.34 |
| Czechia(a,b,c) | H | 8,463,616 | 343 | 86 | 10,099 | 2,470 | 8,462 | 21,032 | 294 | 72 | 211 | 577 | 0.23 |
| Denmark(b) | H | 4,499,620 | 527 | 86 | 22,213 | 3,139 | 42,511 | 67,863 | 526 | 74 | 865 | 1,466 | 0.42 |
| Estonia(a,b,c) | H | 1,036,771 | 310 | 86 | 8,676 | 2,122 | 8,376 | 19,174 | 28 | 7 | 23 | 58 | 0.19 |
| Finland(a,b,c) | H | 4,364,468 | 395 | 81 | 11,799 | 2,886 | 17,390 | 32,075 | 203 | 50 | 243 | 496 | 0.18 |
| France(b) | H | 50,224,101 | 386 | 84 | 15,844 | 3,486 | 14,481 | 33,810 | 3,073 | 676 | 2,368 | 6,117 | 0.22 |
| Germany | H | 69,126,147 | 361 | 85 | 18,615 | 10,784 | 4,876 | 34,276 | 4,646 | 2,692 | 1,034 | 8,372 | 0.22 |
| Greece(a,b,c) | H | 8,365,629 | 393 | 85 | 10,103 | 2,471 | 6,846 | 19,420 | 332 | 81 | 190 | 603 | 0.29 |
| Greenland(c) | H | 40,635 | 525 | 93 |  |  | 19,044 | 19,044 |  |  | 4 | 4 | 0.13 |
| Guam(c) | H | 110,139 | 475 | 91 |  |  | 13,500 | 13,500 |  |  | 6 | 6 | 0.10 |
| Hungary(a,b) | H | 7,799,500 | 332 | 86 | 8,185 | 2,002 | 19,069 | 29,257 | 212 | 52 | 424 | 688 | 0.42 |
| Iceland(a,b,c) | H | 255,664 | 409 | 88 | 11,135 | 2,724 | 24,621 | 38,480 | 12 | 3 | 23 | 37 | 0.15 |
| Ireland(a,b,c) | H | 3,569,511 | 499 | 88 | 8,610 | 2,106 | 28,939 | 39,654 | 154 | 38 | 454 | 646 | 0.16 |
| Israel(a,b,c) | H | 5,997,590 | 407 | 89 | 9,618 | 2,352 | 15,896 | 27,866 | 235 | 57 | 345 | 637 | 0.16 |
| Italy | H | 49,486,721 | 375 | 84 | 9,790 | 1,099 | 12,916 | 23,805 | 1,817 | 204 | 2,005 | 4,026 | 0.20 |
| Japan(b) | H | 106,577,233 | 384 | 83 | 9,727 | 3,387 | 21,355 | 34,469 | 3,985 | 1,388 | 7,244 | 12,617 | 0.25 |
| Kuwait(a,b,c) | H | 3,314,390 | 462 | 98 | 7,084 | 1,733 | 10,966 | 19,784 | 109 | 27 | 164 | 300 | 0.22 |
| Latvia(a,b,c) | H | 1,521,473 | 305 | 86 | 8,479 | 2,074 | 6,417 | 16,970 | 39 | 10 | 26 | 75 | 0.22 |
| Lithuania(a,b,c) | H | 2,240,317 | 304 | 86 | 9,031 | 2,209 | 7,007 | 18,247 | 61 | 15 | 41 | 118 | 0.22 |
| Luxembourg(a,b,c) | H | 486,007 | 427 | 89 | 6,923 | 1,693 | 40,272 | 48,889 | 14 | 4 | 74 | 92 | 0.13 |
| Malta(a,b,c) | H | 356,099 | 400 | 84 | 10,589 | 2,590 | 11,156 | 24,335 | 15 | 4 | 13 | 32 | 0.20 |
| Mauritius(a,b,c) | H | 963,414 | 458 | 92 | 7,991 | 1,955 | 4,079 | 14,024 | 35 | 9 | 16 | 60 | 0.42 |
| Monaco(a,b,c) | H | 30,869 | 427 | 80 | 1,966 | 481 | 71,295 | 73,742 | 0 | 0 | 8 | 8 | 0.11 |
| Nauru(a,b,c) | H | 5,775 | 449 | 96 | 12,609 | 3,084 | 3,499 | 19,192 | 0 | 0 | 0 | 0 | 0.42 |
| Netherlands(a,b,c) | H | 13,388,312 | 549 | 85 | 13,058 | 3,194 | 18,765 | 35,017 | 960 | 235 | 1,175 | 2,371 | 0.26 |
| New Zealand(b,c) | H | 3,709,413 | 560 | 90 | 23,308 | 3,071 | 15,328 | 41,708 | 484 | 64 | 287 | 835 | 0.39 |
| Northern Mariana Islands(c) | H | 33,217 | 497 | 94 |  |  | 8,477 | 8,477 |  |  | 1 | 1 | 0.11 |
| Norway(b) | H | 4,094,087 | 417 | 86 | 61,571 | 3,317 | 9,175 | 74,064 | 1,052 | 57 | 136 | 1,245 | 0.31 |
| Oman(a,b,c) | H | 2,932,287 | 445 | 98 | 5,249 | 1,284 | 6,841 | 13,374 | 68 | 17 | 88 | 173 | 0.20 |
| Palau(a,b,c) | H | 13,471 | 471 | 94 | 19,529 | 4,777 | 5,567 | 29,873 | 1 | 0 | 0 | 2 | 0.67 |
| Panama(a,b,c) | H | 2,662,916 | 382 | 92 | 9,776 | 2,391 | 5,659 | 17,826 | 100 | 24 | 53 | 177 | 0.26 |
| Poland(b,c) | H | 30,716,094 | 344 | 88 | 8,310 | 2,033 | 5,614 | 15,957 | 877 | 214 | 520 | 1,611 | 0.27 |
| Portugal(a,b,c) | H | 8,684,607 | 392 | 84 | 12,286 | 3,005 | 8,343 | 23,634 | 418 | 102 | 239 | 759 | 0.32 |
| Puerto Rico(c) | H | 2,661,886 | 344 | 84 |  |  | 11,771 | 11,771 |  |  | 91 | 91 | 0.09 |
| Qatar(a,b,c) | H | 2,281,174 | 488 | 99 | 3,752 | 918 | 22,398 | 27,068 | 42 | 10 | 247 | 299 | 0.17 |
| Romania(a,b,c) | H | 15,156,701 | 330 | 87 | 7,405 | 1,811 | 4,634 | 13,850 | 371 | 91 | 201 | 662 | 0.26 |
| Saint Kitts and Nevis(a,b,c) | H | 43,319 | 371 | 93 | 6,956 | 1,701 | 8,303 | 16,960 | 1 | 0 | 1 | 3 | 0.24 |
| San Marino(a,b,c) | H | 26,174 | 409 | 85 | 8,237 | 2,015 | 16,909 | 27,161 | 1 | 0 | 2 | 3 | 0.16 |
| Saudi Arabia(a,b,c) | H | 26,003,023 | 440 | 98 | 7,332 | 1,793 | 8,021 | 17,147 | 838 | 205 | 900 | 1,943 | 0.24 |
| Seychelles(a,b,c) | H | 73,281 | 482 | 94 | 6,690 | 1,636 | 6,170 | 14,497 | 2 | 1 | 2 | 5 | 0.30 |
| Singapore(a,b,c) | H | 4,628,467 | 413 | 92 | 5,261 | 1,287 | 23,541 | 30,089 | 101 | 25 | 416 | 541 | 0.14 |
| Slovakia(a,b,c) | H | 4,323,306 | 347 | 89 | 8,970 | 2,194 | 6,931 | 18,095 | 134 | 33 | 92 | 259 | 0.25 |
| Slovenia(a,b,c) | H | 1,670,519 | 339 | 86 | 10,987 | 2,687 | 9,303 | 22,978 | 62 | 15 | 45 | 123 | 0.23 |
| South Korea | H | 42,795,895 | 403 | 91 | 6,581 | 3,939 | 8,053 | 18,573 | 1,134 | 679 | 1,259 | 3,071 | 0.19 |
| Spain(a,b,c) | H | 36,987,625 | 410 | 86 | 11,772 | 2,879 | 10,578 | 25,229 | 1,787 | 437 | 1,388 | 3,612 | 0.26 |
| Sweden | H | 7,857,835 | 421 | 82 | 30,046 | 3,426 | 39,572 | 73,045 | 993 | 113 | 1,072 | 2,179 | 0.41 |
| Switzerland | H | 7,039,431 | 413 | 86 | 13,275 | 1,360 | 25,696 | 40,331 | 386 | 40 | 645 | 1,070 | 0.15 |
| Taiwan (Province of China)(b,c) | H | 19,380,860 | 470 | 89 | 6,583 | 1,986 | 9,263 | 17,832 | 600 | 181 | 754 | 1,534 | 0.25 |
| Trinidad and Tobago(a,b,c) | H | 1,024,183 | 364 | 90 | 9,049 | 2,213 | 5,611 | 16,873 | 34 | 8 | 19 | 61 | 0.26 |
| United Arab Emirates(a,b,c) | H | 7,825,551 | 499 | 99 | 5,511 | 1,348 | 16,226 | 23,085 | 215 | 53 | 629 | 897 | 0.21 |
| United Kingdom(a,b,c) | H | 51,642,381 | 318 | 85 | 13,089 | 3,201 | 15,286 | 31,576 | 2,149 | 526 | 2,132 | 4,806 | 0.17 |
| United States of America | H | 247,761,606 | 526 | 88 | 24,632 | 22,663 | 4,392 | 51,687 | 32,096 | 29,531 | 5,054 | 66,680 | 0.31 |
| Uruguay(a,b,c) | H | 2,478,938 | 414 | 90 | 12,050 | 2,947 | 6,387 | 21,383 | 124 | 30 | 59 | 213 | 0.35 |
| Afghanistan | L | 12,436,632 | 345 | 98 |  |  |  |  |  |  |  |  |  |
| Burkina Faso | L | 9,361,347 | 352 | 97 |  |  |  |  |  |  |  |  |  |
| Burundi | L | 5,496,174 | 331 | 97 |  |  |  |  |  |  |  |  |  |
| Central African Republic | L | 28,631,757 | 428 | 98 |  |  |  |  |  |  |  |  |  |
| Chad | L | 2,470,098 | 321 | 97 |  |  |  |  |  |  |  |  |  |
| Democratic People's Republic of Korea | L | 19,455,460 | 409 | 94 |  |  |  |  |  |  |  |  |  |
| Democratic Republic of the Congo | L | 39,524,964 | 331 | 97 |  |  |  |  |  |  |  |  |  |
| Eritrea | L | 3,197,290 | 345 | 98 |  |  |  |  |  |  |  |  |  |
| Ethiopia | L | 48,667,231 | 350 | 97 |  |  |  |  |  |  |  |  |  |
| Gambia | L | 1,059,891 | 354 | 97 |  |  |  |  |  |  |  |  |  |
| Guinea | L | 5,631,790 | 354 | 96 |  |  |  |  |  |  |  |  |  |
| Guinea-Bissau | L | 898,312 | 350 | 97 |  |  |  |  |  |  |  |  |  |
| Haiti | L | 6,935,805 | 318 | 96 |  |  |  |  |  |  |  |  |  |
| Liberia | L | 2,501,674 | 356 | 98 |  |  |  |  |  |  |  |  |  |
| Madagascar | L | 12,754,861 | 347 | 98 |  |  |  |  |  |  |  |  |  |
| Malawi | L | 8,241,426 | 325 | 97 |  |  |  |  |  |  |  |  |  |
| Mali | L | 9,143,173 | 351 | 97 |  |  |  |  |  |  |  |  |  |
| Mozambique | L | 12,501,170 | 326 | 97 |  |  |  |  |  |  |  |  |  |
| Niger | L | 8,743,029 | 343 | 97 |  |  |  |  |  |  |  |  |  |
| Rwanda | L | 6,436,209 | 346 | 97 |  |  |  |  |  |  |  |  |  |
| Sierra Leone | L | 4,017,187 | 350 | 97 |  |  |  |  |  |  |  |  |  |
| Somalia | L | 8,121,396 | 312 | 98 |  |  |  |  |  |  |  |  |  |
| South Sudan | L | 3,893,333 | 356 | 97 |  |  |  |  |  |  |  |  |  |
| Sudan | L | 20,835,342 | 368 | 97 |  |  |  |  |  |  |  |  |  |
| Syrian Arab Republic | L | 8,224,706 | 381 | 94 |  |  |  |  |  |  |  |  |  |
| Tajikistan | L | 5,440,339 | 333 | 97 |  |  |  |  |  |  |  |  |  |
| Togo | L | 3,958,575 | 359 | 97 |  |  |  |  |  |  |  |  |  |
| Uganda | L | 17,158,449 | 331 | 97 |  |  |  |  |  |  |  |  |  |
| Yemen | L | 15,208,768 | 367 | 97 |  |  |  |  |  |  |  |  |  |
| Algeria(a,b,c) | LM | 27,056,579 | 398 | 95 | 1,810 | 1,007 | 1,600 | 4,417 | 195 | 108 | 164 | 467 | 0.27 |
| Angola(a,b,c) | LM | 13,087,608 | 357 | 97 | 735 | 409 | 852 | 1,995 | 34 | 19 | 39 | 92 | 0.13 |
| Bangladesh(a,b,c) | LM | 99,817,962 | 447 | 94 | 720 | 400 | 844 | 1,965 | 322 | 179 | 355 | 855 | 0.24 |
| Benin(a,b,c) | LM | 5,566,458 | 360 | 97 | 693 | 385 | 466 | 1,544 | 14 | 8 | 9 | 31 | 0.21 |
| Bhutan(a,b,c) | LM | 495,105 | 466 | 95 | 1,046 | 582 | 1,314 | 2,942 | 2 | 1 | 3 | 7 | 0.26 |
| Bolivia (Plurinational State of)(a,b,c) | LM | 7,012,578 | 365 | 94 | 2,008 | 1,116 | 1,381 | 4,505 | 51 | 29 | 33 | 113 | 0.28 |
| Cabo Verde(a,b,c) | LM | 12,819,905 | 363 | 96 | 1,431 | 796 | 1,366 | 3,594 | 2 | 1 | 2 | 5 | 0.24 |
| Cambodia(a,b,c) | LM | 353,888 | 383 | 96 | 2,027 | 1,127 | 665 | 3,819 | 86 | 48 | 27 | 162 | 0.60 |
| Cameroon(a,b,c) | LM | 10,024,555 | 426 | 97 | 1,043 | 580 | 612 | 2,234 | 52 | 29 | 30 | 110 | 0.28 |
| Comoros(a,b,c) | LM | 33,191,979 | 371 | 96 | 1,499 | 834 | 601 | 2,934 | 2 | 1 | 1 | 4 | 0.36 |
| Congo(a,b,c) | LM | 412,801 | 357 | 97 | 604 | 336 | 910 | 1,851 | 6 | 3 | 9 | 18 | 0.14 |
| Côte d'Ivoire(a,b,c) | LM | 3,297,815 | 381 | 97 | 957 | 532 | 891 | 2,379 | 45 | 25 | 40 | 110 | 0.19 |
| Djibouti(a,b,c) | LM | 686,956 | 367 | 97 | 521 | 290 | 1,144 | 1,955 | 1 | 1 | 3 | 5 | 0.16 |
| Egypt(a,b,c) | LM | 56,888,666 | 391 | 96 | 1,374 | 764 | 1,141 | 3,280 | 306 | 170 | 245 | 721 | 0.24 |
| El Salvador(a,b,c) | LM | 3,976,005 | 350 | 93 | 2,078 | 1,156 | 1,703 | 4,936 | 29 | 16 | 22 | 67 | 0.25 |
| Eswatini(a,b,c) | LM | 603,162 | 352 | 96 | 1,966 | 1,093 | 1,529 | 4,589 | 4 | 2 | 3 | 10 | 0.21 |
| Ghana(a,b,c) | LM | 16,918,561 | 371 | 97 | 991 | 551 | 862 | 2,405 | 62 | 35 | 52 | 149 | 0.22 |
| Honduras(a,b,c) | LM | 5,443,229 | 352 | 95 | 2,112 | 1,175 | 1,002 | 4,289 | 41 | 23 | 18 | 81 | 0.32 |
| India | LM | 879,785,395 | 467 | 94 | 893 | 889 | 1,281 | 3,064 | 3,670 | 3,653 | 4,954 | 12,276 | 0.43 |
| Kenya(a,b,c) | LM | 24,072,603 | 355 | 97 | 1,331 | 740 | 784 | 2,856 | 114 | 63 | 65 | 242 | 0.24 |
| Kiribati(a,b,c) | LM | 65,701 | 411 | 97 | 2,976 | 1,655 | 570 | 5,201 | 1 | 0 | 0 | 1 | 0.79 |
| Kyrgyzstan(a,b,c) | LM | 3,928,357 | 332 | 96 | 1,303 | 725 | 547 | 2,575 | 17 | 9 | 7 | 33 | 0.38 |
| Lao People's Democratic Republic(a,b,c) | LM | 4,199,558 | 439 | 96 | 753 | 419 | 1,034 | 2,206 | 14 | 8 | 18 | 40 | 0.21 |
| Lesotho(a,b,c) | LM | 1,016,963 | 327 | 95 | 3,268 | 1,817 | 439 | 5,524 | 11 | 6 | 1 | 18 | 0.75 |
| Mauritania(a,b,c) | LM | 1,953,283 | 367 | 96 | 958 | 533 | 732 | 2,222 | 7 | 4 | 5 | 16 | 0.19 |
| Micronesia (Federated States of)(a,b,c) | LM | 59,990 | 419 | 96 | 3,318 | 1,845 | 1,471 | 6,634 | 1 | 0 | 0 | 2 | 0.40 |
| Mongolia(a,b,c) | LM | 1,973,138 | 351 | 97 | 1,092 | 607 | 1,748 | 3,448 | 8 | 4 | 12 | 23 | 0.17 |
| Morocco(a,b,c) | LM | 23,640,161 | 377 | 95 | 1,539 | 856 | 1,392 | 3,787 | 137 | 76 | 118 | 331 | 0.26 |
| Myanmar(a,b,c) | LM | 34,649,322 | 427 | 95 | 1,357 | 754 | 515 | 2,626 | 201 | 112 | 72 | 385 | 0.56 |
| Nepal(a,b,c) | LM | 17,928,802 | 429 | 94 | 1,289 | 717 | 472 | 2,477 | 99 | 55 | 34 | 189 | 0.55 |
| Nicaragua(a,b,c) | LM | 3,918,641 | 367 | 95 | 2,437 | 1,355 | 752 | 4,544 | 35 | 19 | 10 | 65 | 0.51 |
| Nigeria | LM | 97,167,413 | 392 | 97 | 1,289 | 324 | 617 | 2,231 | 491 | 124 | 228 | 843 | 0.19 |
| Pakistan(a,b,c) | LM | 119,507,877 | 459 | 96 | 980 | 545 | 572 | 2,097 | 537 | 299 | 301 | 1,138 | 0.35 |
| Palestine(c) | LM | 2,584,414 | 386 | 97 |  |  | 1,455 | 1,455 |  |  | 14 | 14 | 0.08 |
| Papua New Guinea(a,b,c) | LM | 5,241,772 | 438 | 97 | 667 | 371 | 1,032 | 2,070 | 15 | 9 | 23 | 47 | 0.19 |
| Philippines(a,b,c) | LM | 65,701,936 | 455 | 96 | 1,182 | 657 | 1,358 | 3,198 | 353 | 196 | 388 | 938 | 0.25 |
| Republic of Moldova(a,b,c) | LM | 2,934,661 | 305 | 91 | 1,849 | 1,028 | 1,787 | 4,665 | 17 | 9 | 15 | 40 | 0.34 |
| Sao Tome and Principe(a,b,c) | LM | 108,404 | 378 | 97 | 1,603 | 892 | 792 | 3,287 | 1 | 0 | 0 | 1 | 0.31 |
| Senegal(a,b,c) | LM | 7,353,166 | 364 | 96 | 1,198 | 666 | 582 | 2,446 | 32 | 18 | 15 | 65 | 0.28 |
| Solomon Islands(a,b,c) | LM | 335,944 | 418 | 97 | 1,384 | 770 | 954 | 3,108 | 2 | 1 | 1 | 4 | 0.27 |
| Sri Lanka(a,b,c) | LM | 15,033,167 | 451 | 92 | 1,183 | 658 | 1,624 | 3,465 | 80 | 45 | 101 | 226 | 0.25 |
| Timor-Leste(a,b,c) | LM | 672,154 | 395 | 94 | 2,076 | 1,154 | 630 | 3,860 | 6 | 3 | 2 | 10 | 0.50 |
| Tunisia(a,b,c) | LM | 8,119,411 | 391 | 94 | 2,018 | 1,122 | 1,383 | 4,523 | 64 | 36 | 41 | 141 | 0.34 |
| Ukraine(a,b,c) | LM | 35,221,355 | 308 | 89 | 2,058 | 1,145 | 1,456 | 4,660 | 223 | 124 | 141 | 488 | 0.32 |
| United Republic of Tanzania(a,b,c) | LM | 25,953,642 | 344 | 97 | 1,110 | 617 | 418 | 2,146 | 99 | 55 | 36 | 190 | 0.31 |
| Uzbekistan(a,b,c) | LM | 20,990,959 | 344 | 96 | 1,629 | 906 | 710 | 3,244 | 117 | 65 | 49 | 232 | 0.39 |
| Vanuatu(a,b,c) | LM | 156,779 | 422 | 96 | 974 | 542 | 1,224 | 2,740 | 1 | 0 | 1 | 2 | 0.19 |
| Viet Nam(a,b,c) | LM | 67,364,721 | 514 | 95 | 1,522 | 847 | 1,389 | 3,758 | 527 | 293 | 458 | 1,279 | 0.38 |
| Zambia(a,b,c) | LM | 8,421,134 | 343 | 97 | 1,540 | 857 | 504 | 2,901 | 44 | 25 | 14 | 83 | 0.36 |
| Zimbabwe(a,b,c) | LM | 7,312,244 | 330 | 97 | 2,234 | 1,242 | 566 | 4,042 | 54 | 30 | 13 | 97 | 0.44 |
| Albania(a,b,c) | UM | 2,055,002 | 328 | 89 | 3,753 | 1,196 | 2,755 | 7,704 | 25 | 8 | 17 | 50 | 0.32 |
| American Samoa(c) | UM | 30,663 | 462 | 94 |  |  | 6,981 | 6,981 |  |  | 1 | 1 | 0.14 |
| Argentina(a,b,c) | UM | 30,963,187 | 425 | 92 | 6,821 | 2,174 | 5,087 | 14,082 | 897 | 286 | 613 | 1,796 | 0.40 |
| Armenia(a,b,c) | UM | 2,257,269 | 339 | 92 | 8,130 | 2,591 | 2,465 | 13,187 | 62 | 20 | 17 | 99 | 0.73 |
| Azerbaijan(a,b,c) | UM | 7,314,945 | 356 | 96 | 2,896 | 923 | 2,454 | 6,273 | 75 | 24 | 61 | 160 | 0.33 |
| Belarus(a,b,c) | UM | 7,412,563 | 313 | 90 | 4,204 | 1,340 | 3,491 | 9,035 | 98 | 31 | 73 | 201 | 0.31 |
| Belize(a,b,c) | UM | 243,910 | 345 | 95 | 4,280 | 1,364 | 3,171 | 8,815 | 4 | 1 | 3 | 7 | 0.30 |
| Bosnia and Herzegovina(a,b,c) | UM | 2,700,795 | 330 | 88 | 6,490 | 2,069 | 3,069 | 11,628 | 58 | 18 | 24 | 100 | 0.50 |
| Botswana(a,b,c) | UM | 1,418,392 | 372 | 96 | 4,338 | 1,383 | 3,410 | 9,131 | 23 | 7 | 17 | 48 | 0.28 |
| Brazil(b,c) | UM | 152,523,561 | 381 | 93 | 3,397 | 2,193 | 4,516 | 10,105 | 1,973 | 1,274 | 2,435 | 5,682 | 0.30 |
| Bulgaria(a,b,c) | UM | 5,630,656 | 326 | 85 | 5,111 | 1,629 | 5,044 | 11,784 | 94 | 30 | 79 | 202 | 0.29 |
| China | UM | 13,504,776 | 433 | 91 | 5,139 | 1,336 | 2,032 | 8,508 | 26,566 | 6,906 | 9,545 | 43,017 | 0.30 |
| Colombia(a,b,c) | UM | 1,084,156,891 | 477 | 92 | 5,529 | 1,762 | 3,287 | 10,579 | 682 | 217 | 375 | 1,274 | 0.39 |
| Costa Rica(a,b,c) | UM | 12,438 | 461 | 93 | 5,217 | 1,663 | 6,468 | 13,348 | 66 | 21 | 75 | 162 | 0.25 |
| Cuba(a,b,c) | UM | 8,875,537 | 346 | 89 | 8,134 | 2,592 | 4,666 | 15,392 | 250 | 80 | 128 | 458 | 0.44 |
| Dominica(a,b,c) | UM | 47,015 | 347 | 91 | 3,916 | 1,248 | 4,371 | 9,536 | 1 | 0 | 1 | 1 | 0.24 |
| Dominican Republic(a,b,c) | UM | 6,901,188 | 352 | 93 | 4,253 | 1,355 | 4,173 | 9,781 | 103 | 33 | 94 | 231 | 0.26 |
| Ecuador(a,b,c) | UM | 10,920,642 | 369 | 93 | 5,607 | 1,787 | 3,182 | 10,577 | 226 | 72 | 119 | 417 | 0.39 |
| Equatorial Guinea(a,b,c) | UM | 681,127 | 368 | 97 | 2,249 | 717 | 3,736 | 6,701 | 6 | 2 | 9 | 17 | 0.15 |
| Fiji(a,b,c) | UM | 569,039 | 457 | 95 | 2,737 | 872 | 3,047 | 6,657 | 7 | 2 | 8 | 17 | 0.31 |
| Gabon(a,b,c) | UM | 945,125 | 367 | 96 | 1,986 | 633 | 3,841 | 6,460 | 7 | 2 | 13 | 22 | 0.13 |
| Georgia(a,b,c) | UM | 2,740,717 | 325 | 89 | 4,775 | 1,522 | 2,398 | 8,695 | 43 | 14 | 19 | 75 | 0.43 |
| Grenada(a,b,c) | UM | 72,120 | 351 | 93 | 3,561 | 1,135 | 5,048 | 9,745 | 1 | 0 | 1 | 2 | 0.20 |
| Guatemala(a,b,c) | UM | 8,698,257 | 351 | 94 | 4,455 | 1,420 | 2,373 | 8,248 | 136 | 43 | 68 | 248 | 0.32 |
| Guyana(a,b,c) | UM | 478,623 | 332 | 94 | 3,536 | 1,127 | 3,307 | 7,971 | 6 | 2 | 5 | 12 | 0.24 |
| Indonesia(b,c) | UM | 181,532,009 | 464 | 95 | 1,062 | 664 | 2,119 | 3,846 | 896 | 560 | 1,704 | 3,160 | 0.28 |
| Iran (Islamic Republic of)(a,b,c) | UM | 58,459,559 | 413 | 96 | 4,813 | 1,534 | 1,674 | 8,021 | 1,163 | 371 | 386 | 1,920 | 0.68 |
| Iraq(a,b,c) | UM | 22,374,212 | 392 | 96 | 3,211 | 1,023 | 2,870 | 7,104 | 282 | 90 | 243 | 614 | 0.26 |
| Jamaica(a,b,c) | UM | 1,947,284 | 343 | 93 | 4,373 | 1,394 | 2,873 | 8,640 | 29 | 9 | 18 | 56 | 0.36 |
| Jordan(a,b,c) | UM | 6,733,515 | 401 | 97 | 5,438 | 1,733 | 2,147 | 9,319 | 147 | 47 | 56 | 250 | 0.56 |
| Kazakhstan(a,b,c) | UM | 12,091,686 | 354 | 94 | 1,999 | 637 | 5,010 | 7,646 | 86 | 27 | 202 | 314 | 0.17 |
| Lebanon(a,b,c) | UM | 3,852,490 | 391 | 94 | 6,202 | 1,977 | 4,588 | 12,766 | 94 | 30 | 65 | 188 | 0.36 |
| Libya(c) | UM | 4,632,152 | 405 | 97 |  |  | 5,382 | 5,382 |  |  | 98 | 98 | 0.14 |
| Malaysia | UM | 20,864,617 | 484 | 94 | 5,262 | 3,733 | 10,096 | 19,091 | 531 | 377 | 963 | 1,872 | 0.51 |
| Maldives(a,b,c) | UM | 357,376 | 520 | 97 | 5,767 | 1,838 | 5,677 | 13,281 | 11 | 3 | 10 | 24 | 0.44 |
| Marshall Islands(a,b,c) | UM | 32,363 | 434 | 97 | 11,721 | 3,736 | 2,649 | 18,106 | 2 | 1 | 0 | 3 | 1.09 |
| Mexico(b,c) | UM | 83,498,254 | 389 | 93 | 4,986 | 1,242 | 5,180 | 11,408 | 1,622 | 404 | 1,574 | 3,600 | 0.28 |
| Montenegro(a,b,c) | UM | 469,408 | 336 | 89 | 5,974 | 1,904 | 4,549 | 12,428 | 9 | 3 | 6 | 19 | 0.34 |
| Namibia(a,b,c) | UM | 1,312,042 | 358 | 96 | 6,094 | 1,943 | 2,617 | 10,654 | 29 | 9 | 12 | 50 | 0.40 |
| North Macedonia(a,b,c) | UM | 1,725,667 | 343 | 90 | 5,203 | 1,658 | 3,099 | 9,961 | 31 | 10 | 17 | 57 | 0.45 |
| Paraguay(a,b,c) | UM | 4,323,335 | 376 | 94 | 5,145 | 1,640 | 2,965 | 9,750 | 84 | 27 | 45 | 156 | 0.41 |
| Peru(a,b,c) | UM | 22,675,948 | 376 | 93 | 3,740 | 1,192 | 3,551 | 8,484 | 319 | 102 | 283 | 703 | 0.31 |
| Russian Federation(a,b,c) | UM | 112,270,523 | 319 | 90 | 4,051 | 1,291 | 5,890 | 11,233 | 1,453 | 463 | 1,890 | 3,806 | 0.22 |
| Saint Lucia(a,b,c) | UM | 132,749 | 352 | 92 | 3,102 | 989 | 5,987 | 10,078 | 1 | 0 | 3 | 4 | 0.21 |
| Saint Vincent and the Grenadines(a,b,c) | UM | 79,096 | 345 | 91 | 3,409 | 1,087 | 4,429 | 8,924 | 1 | 0 | 1 | 2 | 0.26 |
| Samoa(a,b,c) | UM | 109,534 | 439 | 95 | 4,564 | 1,455 | 2,200 | 8,218 | 2 | 1 | 1 | 4 | 0.43 |
| Serbia(a,b,c) | UM | 7,084,226 | 329 | 87 | 6,220 | 1,983 | 3,787 | 11,989 | 145 | 46 | 77 | 268 | 0.52 |
| South Africa(a,b,c) | UM | 36,160,578 | 367 | 95 | 6,533 | 2,082 | 3,415 | 12,031 | 868 | 277 | 431 | 1,575 | 0.41 |
| Suriname(a,b,c) | UM | 382,370 | 310 | 92 | 6,984 | 2,226 | 3,416 | 12,625 | 8 | 3 | 4 | 15 | 0.36 |
| Thailand | UM | 52,428,464 | 445 | 91 | 1,710 | 1,010 | 4,868 | 7,588 | 399 | 235 | 1,034 | 1,668 | 0.31 |
| Tonga(a,b,c) | UM | 56,094 | 440 | 94 | 3,573 | 1,139 | 2,491 | 7,203 | 1 | 0 | 1 | 2 | 0.34 |
| Turkey(a,b,c) | UM | 57,468,633 | 385 | 93 | 3,116 | 993 | 4,648 | 8,756 | 689 | 220 | 961 | 1,870 | 0.25 |
| Turkmenistan(a,b,c) | UM | 3,122,220 | 348 | 96 | 4,711 | 1,502 | 3,750 | 9,963 | 51 | 16 | 39 | 107 | 0.24 |
| Tuvalu(a,b,c) | UM | 7,206 | 420 | 94 | 17,186 | 5,478 | 2,527 | 25,191 | 1 | 0 | 0 | 1 | 1.40 |
| Venezuela (Bolivarian Republic of)(a,b) | UM | 18,794,244 | 381 | 93 | 3,849 | 1,227 |  | 5,076 | 275 | 88 |  | 363 | - |

GNI: Gross national income group; H: high income; UM: upper-middle income; LM: lower-middle income; L: low income; GDP: gross domestic product. Empty cells indicate the absence of viable primary or imputed data for these countries.

Letters in parentheses behind the country names indicate where data are imputed (a: direct costs; b: informal care costs, c: indirect costs).

Medians used for the imputation of missing country data per GNI group are based on the following countries:

- LM: direct: 1,091 (between India 893 and Nigeria 1,289); informal care: 607 (between Nigeria 324 and India 889); indirect care: 949 (between Nigeria 617 and India 1,281)
- UM: direct: 4,192 (between Brazil 3,397 and Mexico 4,986); informal care: 1,336 (China); indirect care: 4,868 (Thailand)
- HI: direct: 16,103 (Canada); informal care: 3,939 (South Korea); indirect care: 15,993 (between Italy 12,916 and Hungary 19,069)

# Figure 3: Boxplot Diagrams on Costs Relative to Gross Domestic Product

Boxplot diagrams showing the distribution of costs expressed as a percentage of GDP in high, upper-middle, and lower-middle income regions.

# Table 8: Parameters Used in Imputation Procedure

| Country | Parameters used for imputing direct and informal care costs | | | | Parameters used for imputing indirect costs | | | GDP in US$ 2019 |
| --- | --- | --- | --- | --- | --- | --- | --- | --- |
|  | World Bank GNI group (2019) | Health Expenditures of country (% of GDP 2019) | Health Expenditures of same GNI group (% of GDP 2019) | Adjustment factor for median of total direct costs (HExp country / Hexp GNI group) | GDP per capita of country (US$ 2019) | GDP per capita of same GNI group (US$ 2019) | Adjustment factor for median of indirect costs (GDP country / GDP GNI group) |  |
| Afghanistan | L | 13.24 | 4.98 | 2.66 | 500.52 | 741.35 | 0.68 | 18,904,490,263 |
| Albania | UM | 5.23 | 5.84 | 0.90 | 5,396.22 | 9,534.01 | 0.57 | 15,401,830,754 |
| Algeria | LM | 6.24 | 3.76 | 1.66 | 4,022.15 | 2,386.49 | 1.69 | 171,767,403,748 |
| American Samoa | UM | 0.00 | 5.84 | 0.00 | 13,672.58 | 9,534.01 | 1.43 | 647,000,000 |
| Andorra | H | 6.71 | 12.49 | 0.54 | 41,327.50 | 44,723.93 | 0.92 | 3,155,065,488 |
| Angola | LM | 2.53 | 3.76 | 0.67 | 2,142.24 | 2,386.49 | 0.90 | 69,309,110,146 |
| Antigua and Barbuda | H | 4.44 | 12.49 | 0.36 | 18,319.46 | 44,723.93 | 0.41 | 1,687,533,333 |
| Argentina | UM | 9.51 | 5.84 | 1.63 | 9,963.67 | 9,534.01 | 1.05 | 447,754,609,219 |
| Armenia | UM | 11.34 | 5.84 | 1.94 | 4,828.51 | 9,534.01 | 0.51 | 13,619,291,361 |
| Australia | H | 9.91 | 12.49 | 0.79 | 54,941.43 | 44,723.93 | 1.23 | 1,392,227,864,397 |
| Austria | H | 10.43 | 12.49 | 0.84 | 50,070.40 | 44,723.93 | 1.12 | 444,621,176,101 |
| Azerbaijan | UM | 4.04 | 5.84 | 0.69 | 4,805.75 | 9,534.01 | 0.50 | 48,174,235,294 |
| Bahamas | H | 5.75 | 12.49 | 0.46 | 32,610.49 | 44,723.93 | 0.73 | 13,192,800,000 |
| Bahrain | H | 4.01 | 12.49 | 0.32 | 25,869.11 | 44,723.93 | 0.58 | 38,653,318,085 |
| Bangladesh | LM | 2.48 | 3.76 | 0.66 | 2,122.08 | 2,386.49 | 0.89 | 351,238,474,245 |
| Barbados | H | 6.30 | 12.49 | 0.50 | 19,002.96 | 44,723.93 | 0.42 | 5,324,250,000 |
| Belarus | UM | 5.86 | 5.84 | 1.00 | 6,837.72 | 9,534.01 | 0.72 | 64,409,647,194 |
| Belgium | H | 10.66 | 12.49 | 0.85 | 46,638.68 | 44,723.93 | 1.04 | 535,830,876,745 |
| Belize | UM | 5.97 | 5.84 | 1.02 | 6,210.57 | 9,534.01 | 0.65 | 2,416,500,000 |
| Benin | LM | 2.39 | 3.76 | 0.63 | 1,170.97 | 2,386.49 | 0.49 | 14,391,686,309 |
| Bermuda | H |  | 12.49 |  | 116,153.17 | 44,723.93 | 2.60 | 7,423,465,000 |
| Bhutan | LM | 3.61 | 3.76 | 0.96 | 3,303.96 | 2,386.49 | 1.38 | 2,535,657,069 |
| Bolivia (Plurinational State of) | LM | 6.92 | 3.76 | 1.84 | 3,472.38 | 2,386.49 | 1.46 | 40,895,322,844 |
| Bosnia and Herzegovina | UM | 9.05 | 5.84 | 1.55 | 6,011.37 | 9,534.01 | 0.63 | 20,202,479,538 |
| Botswana | UM | 6.05 | 5.84 | 1.03 | 6,679.17 | 9,534.01 | 0.70 | 16,695,928,793 |
| Brazil | UM | 9.59 | 5.84 | 1.64 | 8,845.26 | 9,534.01 | 0.93 | 1,873,274,432,754 |
| Brunei Darussalam | H | 2.16 | 12.49 | 0.17 | 30,748.74 | 44,723.93 | 0.69 | 13,469,422,959 |
| Bulgaria | UM | 7.13 | 5.84 | 1.22 | 9,879.33 | 9,534.01 | 1.04 | 68,915,875,215 |
| Burkina Faso | L | 5.46 | 4.98 | 1.10 | 772.17 | 741.35 | 1.04 | 16,178,162,030 |
| Burundi | L | 7.99 | 4.98 | 1.61 | 216.97 | 741.35 | 0.29 | 2,576,518,846 |
| Côte d'Ivoire | LM | 3.30 | 3.76 | 0.88 | 2,238.81 | 2,386.49 | 0.94 | 58,539,424,930 |
| Cabo Verde | LM | 4.94 | 3.76 | 1.31 | 3,434.56 | 2,386.49 | 1.44 | 1,981,845,741 |
| Cambodia | LM | 6.99 | 3.76 | 1.86 | 1,671.39 | 2,386.49 | 0.70 | 27,089,389,787 |
| Cameroon | LM | 3.60 | 3.76 | 0.96 | 1,538.69 | 2,386.49 | 0.64 | 39,670,977,333 |
| Canada | H | 10.84 | 12.49 | 0.87 | 46,328.67 | 44,723.93 | 1.04 | 1,742,015,045,482 |
| Central African Republic | L | 7.75 | 4.98 | 1.56 | 426.41 | 741.35 | 0.58 | 2,221,301,401 |
| Chad | L | 4.35 | 4.98 | 0.88 | 701.62 | 741.35 | 0.95 | 11,314,951,343 |
| Chile | H | 9.33 | 12.49 | 0.75 | 14,631.95 | 44,723.93 | 0.33 | 278,584,733,103 |
| China | UM | 5.35 | 5.84 | 0.92 | 10,143.84 | 9,534.01 | 1.06 | 14,279,937,500,607 |
| Colombia | UM | 7.71 | 5.84 | 1.32 | 6,438.06 | 9,534.01 | 0.68 | 323,109,540,252 |
| Comoros | LM | 5.17 | 3.76 | 1.37 | 1,510.80 | 2,386.49 | 0.63 | 1,195,019,559 |
| Congo | LM | 2.08 | 3.76 | 0.55 | 2,288.81 | 2,386.49 | 0.96 | 12,750,339,023 |
| Cook Islands |  |  |  |  |  |  |  |  |
| Costa Rica | UM | 7.27 | 5.84 | 1.24 | 12,669.34 | 9,534.01 | 1.33 | 64,417,670,083 |
| Croatia | H | 6.98 | 12.49 | 0.56 | 15,331.88 | 44,723.93 | 0.34 | 62,327,983,580 |
| Cuba | UM | 11.34 | 5.84 | 1.94 | 9,139.42 | 9,534.01 | 0.96 | 103,428,000,000 |
| Cyprus | H | 7.01 | 12.49 | 0.56 | 29,417.14 | 44,723.93 | 0.66 | 25,944,504,646 |
| Czechia | H | 7.83 | 12.49 | 0.63 | 23,664.85 | 44,723.93 | 0.53 | 252,548,179,965 |
| Democratic People's Republic of Korea | L |  | 4.98 |  |  | 741.35 |  |  |
| Democratic Republic of the Congo | L | 3.54 | 4.98 | 0.71 | 575.88 | 741.35 | 0.78 | 51,775,830,726 |
| Denmark | H | 9.96 | 12.49 | 0.80 | 59,592.98 | 44,723.93 | 1.33 | 346,498,737,962 |
| Djibouti | LM | 1.80 | 3.76 | 0.48 | 2,876.04 | 2,386.49 | 1.21 | 3,088,853,639 |
| Dominica | UM | 5.46 | 5.84 | 0.93 | 8,561.59 | 9,534.01 | 0.90 | 611,537,037 |
| Dominican Republic | UM | 5.93 | 5.84 | 1.01 | 8,173.34 | 9,534.01 | 0.86 | 88,941,299,734 |
| Ecuador | UM | 7.82 | 5.84 | 1.34 | 6,233.26 | 9,534.01 | 0.65 | 108,108,009,000 |
| Egypt | LM | 4.74 | 3.76 | 1.26 | 2,869.58 | 2,386.49 | 1.20 | 303,080,865,604 |
| El Salvador | LM | 7.17 | 3.76 | 1.90 | 4,280.29 | 2,386.49 | 1.79 | 26,881,140,000 |
| Equatorial Guinea | UM | 3.14 | 5.84 | 0.54 | 7,317.39 | 9,534.01 | 0.77 | 11,364,133,802 |
| Eritrea | L | 4.46 | 4.98 | 0.90 | 0.00 | 741.35 | 0.00 | 0 |
| Estonia | H | 6.73 | 12.49 | 0.54 | 23,424.48 | 44,723.93 | 0.52 | 31,081,901,909 |
| Eswatini | LM | 6.78 | 3.76 | 1.80 | 3,843.38 | 2,386.49 | 1.61 | 4,495,264,161 |
| Ethiopia | L | 3.24 | 4.98 | 0.65 | 840.45 | 741.35 | 1.13 | 95,912,590,628 |
| Fiji | UM | 3.82 | 5.84 | 0.65 | 5,968.30 | 9,534.01 | 0.63 | 5,481,675,173 |
| Finland | H | 9.15 | 12.49 | 0.73 | 48,629.86 | 44,723.93 | 1.09 | 268,514,916,973 |
| France | H | 11.06 | 12.49 | 0.89 | 40,494.90 | 44,723.93 | 0.91 | 2,728,870,246,706 |
| Gabon | UM | 2.77 | 5.84 | 0.47 | 7,523.86 | 9,534.01 | 0.79 | 16,874,405,460 |
| Gambia | L | 3.82 | 4.98 | 0.77 | 722.87 | 741.35 | 0.98 | 1,813,608,280 |
| Georgia | UM | 6.66 | 5.84 | 1.14 | 4,696.15 | 9,534.01 | 0.49 | 17,470,436,259 |
| Germany | H | 11.70 | 12.49 | 0.94 | 46,793.69 | 44,723.93 | 1.05 | 3,888,226,035,922 |
| Ghana | LM | 3.42 | 3.76 | 0.91 | 2,167.91 | 2,386.49 | 0.91 | 68,337,537,816 |
| Greece | H | 7.84 | 12.49 | 0.63 | 19,144.28 | 44,723.93 | 0.43 | 205,257,014,893 |
| Greenland | H | 0.00 | 12.49 | 0.00 | 53,256.24 | 44,723.93 | 1.19 | 2,994,332,324 |
| Grenada | UM | 4.97 | 5.84 | 0.85 | 9,887.92 | 9,534.01 | 1.04 | 1,213,485,185 |
| Guam | H | 0.00 | 12.49 | 0.00 | 37,752.63 | 44,723.93 | 0.84 | 6,366,000,000 |
| Guatemala | UM | 6.21 | 5.84 | 1.06 | 4,647.69 | 9,534.01 | 0.49 | 77,170,421,168 |
| Guinea | L | 3.98 | 4.98 | 0.80 | 1,043.90 | 741.35 | 1.41 | 13,442,861,444 |
| Guinea-Bissau | L | 8.35 | 4.98 | 1.68 | 730.61 | 741.35 | 0.99 | 1,439,638,443 |
| Guyana | UM | 4.93 | 5.84 | 0.84 | 6,477.30 | 9,534.01 | 0.68 | 5,173,760,192 |
| Haiti | L | 4.73 | 4.98 | 0.95 | 1,324.84 | 741.35 | 1.79 | 14,785,839,383 |
| Honduras | LM | 7.28 | 3.76 | 1.94 | 2,519.37 | 2,386.49 | 1.06 | 25,089,976,947 |
| Hungary | H | 6.35 | 12.49 | 0.51 | 16,782.95 | 44,723.93 | 0.38 | 163,988,594,922 |
| Iceland | H | 8.64 | 12.49 | 0.69 | 68,853.72 | 44,723.93 | 1.54 | 24,826,102,120 |
| India | LM | 3.01 | 3.76 | 0.80 | 2,047.23 | 2,386.49 | 0.86 | 2,831,552,222,520 |
| Indonesia | UM | 2.90 | 5.84 | 0.50 | 4,151.23 | 9,534.01 | 0.44 | 1,119,099,868,265 |
| Iran (Islamic Republic of) | UM | 6.71 | 5.84 | 1.15 | 3,277.88 | 9,534.01 | 0.34 | 283,746,685,180 |
| Iraq | UM | 4.48 | 5.84 | 0.77 | 5,621.18 | 9,534.01 | 0.59 | 233,636,097,800 |
| Ireland | H | 6.68 | 12.49 | 0.53 | 80,927.07 | 44,723.93 | 1.81 | 399,321,701,632 |
| Israel | H | 7.46 | 12.49 | 0.60 | 44,452.23 | 44,723.93 | 0.99 | 402,470,513,619 |
| Italy | H | 8.67 | 12.49 | 0.69 | 33,673.75 | 44,723.93 | 0.75 | 2,011,302,198,827 |
| Jamaica | UM | 6.10 | 5.84 | 1.04 | 5,626.17 | 9,534.01 | 0.59 | 15,830,768,550 |
| Japan | H | 10.74 | 12.49 | 0.86 | 40,458.00 | 44,723.93 | 0.90 | 5,123,318,151,511 |
| Jordan | UM | 7.58 | 5.84 | 1.30 | 4,205.56 | 9,534.01 | 0.44 | 44,993,992,365 |
| Kazakhstan | UM | 2.79 | 5.84 | 0.48 | 9,812.60 | 9,534.01 | 1.03 | 181,667,190,076 |
| Kenya | LM | 4.59 | 3.76 | 1.22 | 1,970.11 | 2,386.49 | 0.83 | 100,379,713,697 |
| Kiribati | LM | 10.26 | 3.76 | 2.73 | 1,432.18 | 2,386.49 | 0.60 | 177,935,349 |
| Kuwait | H | 5.50 | 12.49 | 0.44 | 30,667.35 | 44,723.93 | 0.69 | 136,196,760,181 |
| Kyrgyzstan | LM | 4.49 | 3.76 | 1.19 | 1,374.03 | 2,386.49 | 0.58 | 8,871,026,074 |
| Lao People's Democratic Republic | LM | 2.60 | 3.76 | 0.69 | 2,598.51 | 2,386.49 | 1.09 | 18,740,559,554 |
| Latvia | H | 6.58 | 12.49 | 0.53 | 17,945.22 | 44,723.93 | 0.40 | 34,343,961,073 |
| Lebanon | UM | 8.65 | 5.84 | 1.48 | 8,985.57 | 9,534.01 | 0.94 | 51,953,744,530 |
| Lesotho | LM | 11.27 | 3.76 | 2.99 | 1,102.56 | 2,386.49 | 0.46 | 2,453,980,835 |
| Liberia | L | 8.47 | 4.98 | 1.70 | 665.88 | 741.35 | 0.90 | 3,319,596,500 |
| Libya | UM | 0.00 | 5.84 | 0.00 | 10,542.15 | 9,534.01 | 1.11 | 69,252,306,372 |
| Lithuania | H | 7.01 | 12.49 | 0.56 | 19,595.14 | 44,723.93 | 0.44 | 54,751,510,088 |
| Luxembourg | H | 5.37 | 12.49 | 0.43 | 112,621.82 | 44,723.93 | 2.52 | 69,825,641,851 |
| Madagascar | L | 3.69 | 4.98 | 0.74 | 512.28 | 741.35 | 0.69 | 14,104,664,515 |
| Malawi | L | 7.39 | 4.98 | 1.49 | 584.36 | 741.35 | 0.79 | 11,025,371,147 |
| Malaysia | UM | 3.83 | 5.84 | 0.65 | 11,132.02 | 9,534.01 | 1.17 | 365,175,135,788 |
| Maldives | UM | 8.04 | 5.84 | 1.38 | 11,118.56 | 9,534.01 | 1.17 | 5,609,400,722 |
| Mali | L | 3.89 | 4.98 | 0.78 | 840.18 | 741.35 | 1.13 | 17,280,251,194 |
| Malta | H | 8.21 | 12.49 | 0.66 | 31,198.24 | 44,723.93 | 0.70 | 15,725,847,979 |
| Marshall Islands | UM | 16.34 | 5.84 | 2.80 | 5,188.97 | 9,534.01 | 0.54 | 232,092,300 |
| Mauritania | LM | 3.30 | 3.76 | 0.88 | 1,839.96 | 2,386.49 | 0.77 | 8,066,126,312 |
| Mauritius | H | 6.20 | 12.49 | 0.50 | 11,405.73 | 44,723.93 | 0.26 | 14,436,353,898 |
| Mexico | UM | 5.43 | 5.84 | 0.93 | 10,145.17 | 9,534.01 | 1.06 | 1,269,011,767,479 |
| Micronesia (Federated States of) | LM | 11.44 | 3.76 | 3.04 | 3,699.08 | 2,386.49 | 1.55 | 412,000,000 |
| Monaco | H | 1.53 | 12.49 | 0.12 | 199,377.48 | 44,723.93 | 4.46 | 7,383,745,662 |
| Mongolia | LM | 3.77 | 3.76 | 1.00 | 4,394.95 | 2,386.49 | 1.84 | 14,206,359,007 |
| Montenegro | UM | 8.33 | 5.84 | 1.43 | 8,909.65 | 9,534.01 | 0.93 | 5,542,053,845 |
| Morocco | LM | 5.31 | 3.76 | 1.41 | 3,498.57 | 2,386.49 | 1.47 | 128,919,944,682 |
| Mozambique | L | 7.83 | 4.98 | 1.57 | 508.16 | 741.35 | 0.69 | 15,390,039,242 |
| Myanmar | LM | 4.68 | 3.76 | 1.24 | 1,295.20 | 2,386.49 | 0.54 | 68,697,759,361 |
| Namibia | UM | 8.50 | 5.84 | 1.45 | 5,126.18 | 9,534.01 | 0.54 | 12,541,928,105 |
| Nauru | H | 9.78 | 12.49 | 0.78 | 9,786.03 | 44,723.93 | 0.22 | 118,724,074 |
| Nepal | LM | 4.45 | 3.76 | 1.18 | 1,185.68 | 2,386.49 | 0.50 | 34,186,190,996 |
| Netherlands | H | 10.13 | 12.49 | 0.81 | 52,476.27 | 44,723.93 | 1.17 | 910,194,347,569 |
| New Zealand | H | 9.74 | 12.49 | 0.78 | 42,865.23 | 44,723.93 | 0.96 | 213,434,571,358 |
| Nicaragua | LM | 8.40 | 3.76 | 2.23 | 1,890.27 | 2,386.49 | 0.79 | 12,596,636,042 |
| Niger | L | 5.67 | 4.98 | 1.14 | 550.96 | 741.35 | 0.74 | 12,916,455,161 |
| Nigeria | LM | 3.03 | 3.76 | 0.80 | 2,204.18 | 2,386.49 | 0.92 | 448,120,015,210 |
| Niue |  |  |  |  |  |  |  |  |
| North Macedonia | UM | 7.25 | 5.84 | 1.24 | 6,070.39 | 9,534.01 | 0.64 | 12,606,338,449 |
| Northern Mariana Islands | H |  | 12.49 |  | 23,707.33 | 44,723.93 | 0.53 | 1,182,000,000 |
| Norway | H | 10.52 | 12.49 | 0.84 | 75,719.75 | 44,723.93 | 1.69 | 404,941,363,636 |
| Oman | H | 4.07 | 12.49 | 0.33 | 19,132.15 | 44,723.93 | 0.43 | 88,060,858,257 |
| Pakistan | LM | 3.38 | 3.76 | 0.90 | 1,437.17 | 2,386.49 | 0.60 | 320,909,489,230 |
| Palau | H | 15.15 | 12.49 | 1.21 | 15,567.09 | 44,723.93 | 0.35 | 278,900,000 |
| Palestine | LM | 0.00 | 3.76 | 0.00 | 3,656.86 | 2,386.49 | 1.53 | 17,133,500,000 |
| Panama | H | 7.58 | 12.49 | 0.61 | 15,826.08 | 44,723.93 | 0.35 | 66,984,400,000 |
| Papua New Guinea | LM | 2.30 | 3.76 | 0.61 | 2,593.80 | 2,386.49 | 1.09 | 24,751,344,561 |
| Paraguay | UM | 7.17 | 5.84 | 1.23 | 5,807.84 | 9,534.01 | 0.61 | 37,925,338,329 |
| Peru | UM | 5.22 | 5.84 | 0.89 | 6,955.81 | 9,534.01 | 0.73 | 228,323,495,041 |
| Philippines | LM | 4.08 | 3.76 | 1.08 | 3,413.85 | 2,386.49 | 1.43 | 376,823,278,561 |
| Poland | H | 6.45 | 12.49 | 0.52 | 15,699.91 | 44,723.93 | 0.35 | 596,054,591,863 |
| Portugal | H | 9.53 | 12.49 | 0.76 | 23,330.82 | 44,723.93 | 0.52 | 239,986,922,639 |
| Puerto Rico | H | 0.00 | 12.49 | 0.00 | 32,916.87 | 44,723.93 | 0.74 | 105,126,400,000 |
| Qatar | H | 2.91 | 12.49 | 0.23 | 62,637.28 | 44,723.93 | 1.40 | 175,837,550,989 |
| Republic of Moldova | LM | 6.38 | 3.76 | 1.69 | 4,493.37 | 2,386.49 | 1.88 | 11,971,345,003 |
| Romania | H | 5.74 | 12.49 | 0.46 | 12,958.08 | 44,723.93 | 0.29 | 251,019,278,416 |
| Russian Federation | UM | 5.65 | 5.84 | 0.97 | 11,536.25 | 9,534.01 | 1.21 | 1,693,113,904,263 |
| Rwanda | L | 6.41 | 4.98 | 1.29 | 806.88 | 741.35 | 1.09 | 10,356,327,149 |
| Saint Kitts and Nevis | H | 5.40 | 12.49 | 0.43 | 23,219.33 | 44,723.93 | 0.52 | 1,107,840,741 |
| Saint Lucia | UM | 4.32 | 5.84 | 0.74 | 11,726.77 | 9,534.01 | 1.23 | 2,094,201,725 |
| Saint Vincent and the Grenadines | UM | 4.75 | 5.84 | 0.81 | 8,674.37 | 9,534.01 | 0.91 | 910,149,704 |
| Samoa | UM | 6.36 | 5.84 | 1.09 | 4,308.27 | 9,534.01 | 0.45 | 912,944,538 |
| San Marino | H | 6.39 | 12.49 | 0.51 | 47,287.40 | 44,723.93 | 1.06 | 1,616,188,702 |
| Sao Tome and Principe | LM | 5.53 | 3.76 | 1.47 | 1,991.74 | 2,386.49 | 0.83 | 427,425,040 |
| Saudi Arabia | H | 5.69 | 12.49 | 0.46 | 22,430.24 | 44,723.93 | 0.50 | 803,616,264,791 |
| Senegal | LM | 4.13 | 3.76 | 1.10 | 1,462.35 | 2,386.49 | 0.61 | 23,398,811,424 |
| Serbia | UM | 8.67 | 5.84 | 1.48 | 7,417.20 | 9,534.01 | 0.78 | 51,514,222,382 |
| Seychelles | H | 5.19 | 12.49 | 0.42 | 17,253.51 | 44,723.93 | 0.39 | 1,684,373,490 |
| Sierra Leone | L | 8.75 | 4.98 | 1.76 | 506.61 | 741.35 | 0.68 | 4,076,578,543 |
| Singapore | H | 4.08 | 12.49 | 0.33 | 65,831.19 | 44,723.93 | 1.47 | 375,472,731,271 |
| Slovakia | H | 6.96 | 12.49 | 0.56 | 19,383.48 | 44,723.93 | 0.43 | 105,720,354,989 |
| Slovenia | H | 8.52 | 12.49 | 0.68 | 26,016.08 | 44,723.93 | 0.58 | 54,331,588,482 |
| Solomon Islands | LM | 4.77 | 3.76 | 1.27 | 2,398.77 | 2,386.49 | 1.01 | 1,619,154,819 |
| Somalia | L |  | 4.98 |  | 405.79 | 741.35 | 0.55 | 6,485,000,000 |
| South Africa | UM | 9.11 | 5.84 | 1.56 | 6,688.79 | 9,534.01 | 0.70 | 388,531,954,111 |
| South Korea | H | 8.16 | 12.49 | 0.65 | 31,902.42 | 44,723.93 | 0.71 | 1,651,422,932,448 |
| South Sudan | L | 6.04 | 4.98 | 1.21 |  | 741.35 |  |  |
| Spain | H | 9.13 | 12.49 | 0.73 | 29,581.52 | 44,723.93 | 0.66 | 1,394,320,055,129 |
| Sri Lanka | LM | 4.08 | 3.76 | 1.08 | 4,082.69 | 2,386.49 | 1.71 | 89,014,990,792 |
| Sudan | L | 4.57 | 4.98 | 0.92 | 748.01 | 741.35 | 1.01 | 32,338,079,165 |
| Suriname | UM | 9.74 | 5.84 | 1.67 | 6,690.04 | 9,534.01 | 0.70 | 4,016,040,575 |
| Sweden | H | 10.87 | 12.49 | 0.87 | 51,939.43 | 44,723.93 | 1.16 | 533,879,529,188 |
| Switzerland | H | 11.29 | 12.49 | 0.90 | 84,121.93 | 44,723.93 | 1.88 | 721,369,112,727 |
| Syrian Arab Republic | L |  | 4.98 |  | 1,116.68 | 741.35 | 1.51 | 22,443,299,775 |
| Taiwan (Province of China) | H | 6.30 | 12.49 | 0.50 | 25,903.17 | 44,723.93 | 0.58 | 611,396,000,000 |
| Tajikistan | L | 7.11 | 4.98 | 1.43 | 889.02 | 741.35 | 1.20 | 8,300,784,857 |
| Thailand | UM | 3.79 | 5.84 | 0.65 | 7,630.04 | 9,534.01 | 0.80 | 544,081,056,185 |
| Timor-Leste | LM | 7.16 | 3.76 | 1.90 | 1,584.26 | 2,386.49 | 0.66 | 2,028,551,628 |
| Togo | L | 5.73 | 4.98 | 1.15 | 875.93 | 741.35 | 1.18 | 7,220,395,248 |
| Tokelau |  |  |  |  |  |  |  |  |
| Tonga | UM | 4.98 | 5.84 | 0.85 | 4,879.02 | 9,534.01 | 0.51 | 512,057,749 |
| Trinidad and Tobago | H | 7.02 | 12.49 | 0.56 | 15,690.96 | 44,723.93 | 0.35 | 23,849,557,812 |
| Tunisia | LM | 6.96 | 3.76 | 1.85 | 3,477.88 | 2,386.49 | 1.46 | 41,906,113,686 |
| Turkey | UM | 4.34 | 5.84 | 0.74 | 9,103.04 | 9,534.01 | 0.95 | 759,937,390,497 |
| Turkmenistan | UM | 6.57 | 5.84 | 1.12 | 7,344.65 | 9,534.01 | 0.77 | 45,231,428,571 |
| Tuvalu | UM | 23.96 | 5.84 | 4.10 | 4,949.17 | 9,534.01 | 0.52 | 54,223,149 |
| Uganda | L | 3.83 | 4.98 | 0.77 | 823.14 | 741.35 | 1.11 | 35,353,060,634 |
| Ukraine | LM | 7.10 | 3.76 | 1.89 | 3,661.46 | 2,386.49 | 1.53 | 153,882,982,016 |
| United Arab Emirates | H | 4.28 | 12.49 | 0.34 | 45,376.17 | 44,723.93 | 1.01 | 417,989,721,734 |
| United Kingdom | H | 10.15 | 12.49 | 0.81 | 42,747.08 | 44,723.93 | 0.96 | 2,857,057,847,953 |
| United Republic of Tanzania | LM | 3.83 | 3.76 | 1.02 | 1,052.02 | 2,386.49 | 0.44 | 61,136,873,692 |
| United States of America | H | 16.77 | 12.49 | 1.34 | 65,120.39 | 44,723.93 | 1.46 | 21,380,976,119,000 |
| Uruguay | H | 9.35 | 12.49 | 0.75 | 17,859.93 | 44,723.93 | 0.40 | 61,231,149,881 |
| Uzbekistan | LM | 5.62 | 3.76 | 1.49 | 1,784.01 | 2,386.49 | 0.75 | 59,907,674,027 |
| Vanuatu | LM | 3.36 | 3.76 | 0.89 | 3,076.59 | 2,386.49 | 1.29 | 936,526,268 |
| Venezuela (Bolivarian Republic of) | UM | 5.37 | 5.84 | 0.92 |  | 9,534.01 |  |  |
| Viet Nam | LM | 5.25 | 3.76 | 1.40 | 3,491.09 | 2,386.49 | 1.46 | 334,365,257,437 |
| Yemen | L |  | 4.98 |  |  | 741.35 |  |  |
| Zambia | LM | 5.31 | 3.76 | 1.41 | 1,268.12 | 2,386.49 | 0.53 | 23,308,667,781 |
| Zimbabwe | LM | 7.70 | 3.76 | 2.05 | 1,421.87 | 2,386.49 | 0.60 | 21,832,234,926 |

Empty cells indicate that these parameters where not available at the time of analysis.

# Table 9: Supplementary Analysis to Explore Correlations Between Societal Costs, GDP, and Prevalence

| Country | GNI group | Total costs  (million US$ 2019) | GDP  (US$ 2019) | Prevalent cases |
| --- | --- | --- | --- | --- |
| Afghanistan | L |  | 18,904,490,263 | 42,891 |
| Albania | UM | 50 | 15,401,830,754 | 6,741 |
| Algeria | LM | 467 | 171,767,403,748 | 107,603 |
| American Samoa | UM | 1 | 647,000,000 | 142 |
| Andorra | H | 7 | 3,155,065,488 | 297 |
| Angola | LM | 92 | 69,309,110,146 | 46,733 |
| Antigua and Barbuda | H | 3 | 1,687,533,333 | 237 |
| Argentina | UM | 1,796 | 447,754,609,219 | 131,491 |
| Armenia | UM | 99 | 13,619,291,361 | 7,653 |
| Australia | H | 3,588 | 1,392,227,864,397 | 107,460 |
| Austria | H | 932 | 444,621,176,101 | 28,965 |
| Azerbaijan | UM | 160 | 48,174,235,294 | 26,015 |
| Bahamas | H | 20 | 13,192,800,000 | 971 |
| Bahrain | H | 75 | 38,653,318,085 | 4,853 |
| Bangladesh | LM | 855 | 351,238,474,245 | 446,626 |
| Barbados | H | 14 | 5,324,250,000 | 897 |
| Belarus | UM | 201 | 64,409,647,194 | 23,222 |
| Belgium | H | 1,107 | 535,830,876,745 | 35,310 |
| Belize | UM | 7 | 2,416,500,000 | 841 |
| Benin | LM | 31 | 14,391,686,309 | 20,029 |
| Bermuda | H | 7 | 7,423,465,000 | 193 |
| Bhutan | LM | 7 | 2,535,657,069 | 2,305 |
| Bolivia (Plurinational State of) | LM | 113 | 40,895,322,844 | 25,578 |
| Bosnia and Herzegovina | UM | 100 | 20,202,479,538 | 8,908 |
| Botswana | UM | 48 | 16,695,928,793 | 5,278 |
| Brazil | UM | 5,682 | 1,873,274,432,754 | 580,915 |
| Brunei Darussalam | H | 19 | 13,469,422,959 | 1,330 |
| Bulgaria | UM | 202 | 68,915,875,215 | 18,334 |
| Burkina Faso | L |  | 16,178,162,030 | 32,926 |
| Burundi | L |  | 2,576,518,846 | 18,179 |
| Côte d'Ivoire | LM | 110 | 58,539,424,930 | 46,584 |
| Cabo Verde | LM | 5 | 1,981,845,741 | 1,354 |
| Cambodia | LM | 162 | 27,089,389,787 | 42,668 |
| Cameroon | LM | 110 | 39,670,977,333 | 49,738 |
| Canada | H | 4,165 | 1,742,015,045,482 | 122,580 |
| Central African Republic | L |  | 2,221,301,401 | 7,920 |
| Chad | L |  | 11,314,951,343 | 22,197 |
| Chile | H | 1,156 | 278,584,733,103 | 58,465 |
| China | UM | 43,017 | 14,279,937,500,607 | 5,169,074 |
| Colombia | UM | 1,274 | 323,109,540,252 | 123,289 |
| Comoros | LM | 4 | 1,195,019,559 | 1,472 |
| Congo | LM | 18 | 12,750,339,023 | 9,885 |
| Cook Islands |  |  |  | 57 |
| Costa Rica | UM | 162 | 64,417,670,083 | 12,568 |
| Croatia | H | 180 | 62,327,983,580 | 11,301 |
| Cuba | UM | 458 | 103,428,000,000 | 30,744 |
| Cyprus | H | 89 | 25,944,504,646 | 4,325 |
| Czechia | H | 577 | 252,548,179,965 | 29,063 |
| Democratic People's Republic of Korea | L |  |  | 79,599 |
| Democratic Republic of the Congo | L |  | 51,775,830,726 | 130,703 |
| Denmark | H | 1,466 | 346,498,737,962 | 23,692 |
| Djibouti | LM | 5 | 3,088,853,639 | 2,524 |
| Dominica | UM | 1 | 611,537,037 | 163 |
| Dominican Republic | UM | 231 | 88,941,299,734 | 24,263 |
| Ecuador | UM | 417 | 108,108,009,000 | 40,279 |
| Egypt | LM | 721 | 303,080,865,604 | 222,632 |
| El Salvador | LM | 67 | 26,881,140,000 | 13,901 |
| Equatorial Guinea | UM | 17 | 11,364,133,802 | 2,509 |
| Eritrea | L |  |  | 11,038 |
| Estonia | H | 58 | 31,081,901,909 | 3,210 |
| Eswatini | LM | 10 | 4,495,264,161 | 2,121 |
| Ethiopia | L |  | 95,912,590,628 | 170,114 |
| Fiji | UM | 17 | 5,481,675,173 | 2,603 |
| Finland | H | 496 | 268,514,916,973 | 17,220 |
| France | H | 6,117 | 2,728,870,246,706 | 193,948 |
| Gabon | UM | 22 | 16,874,405,460 | 3,464 |
| Gambia | L |  | 1,813,608,280 | 3,751 |
| Georgia | UM | 75 | 17,470,436,259 | 8,906 |
| Germany | H | 8,372 | 3,888,226,035,922 | 249,596 |
| Ghana | LM | 149 | 68,337,537,816 | 62,789 |
| Greece | H | 603 | 205,257,014,893 | 32,855 |
| Greenland | H | 4 | 2,994,332,324 | 213 |
| Grenada | UM | 2 | 1,213,485,185 | 253 |
| Guam | H | 6 | 6,366,000,000 | 523 |
| Guatemala | UM | 248 | 77,170,421,168 | 30,545 |
| Guinea | L |  | 13,442,861,444 | 19,944 |
| Guinea-Bissau | L |  | 1,439,638,443 | 3,147 |
| Guyana | UM | 12 | 5,173,760,192 | 1,590 |
| Haiti | L |  | 14,785,839,383 | 22,043 |
| Honduras | LM | 81 | 25,089,976,947 | 19,184 |
| Hungary | H | 688 | 163,988,594,922 | 25,868 |
| Iceland | H | 37 | 24,826,102,120 | 1,046 |
| India | LM | 12,276 | 2,831,552,222,520 | 4,107,399 |
| Indonesia | UM | 3,160 | 1,119,099,868,265 | 843,059 |
| Iran (Islamic Republic of) | UM | 1,920 | 283,746,685,180 | 241,591 |
| Iraq | UM | 614 | 233,636,097,800 | 87,715 |
| Ireland | H | 646 | 399,321,701,632 | 17,829 |
| Israel | H | 637 | 402,470,513,619 | 24,402 |
| Italy | H | 4,026 | 2,011,302,198,827 | 185,560 |
| Jamaica | UM | 56 | 15,830,768,550 | 6,687 |
| Japan | H | 12,617 | 5,123,318,151,511 | 409,735 |
| Jordan | UM | 250 | 44,993,992,365 | 27,003 |
| Kazakhstan | UM | 314 | 181,667,190,076 | 42,774 |
| Kenya | LM | 242 | 100,379,713,697 | 85,524 |
| Kiribati | LM | 1 | 177,935,349 | 270 |
| Kuwait | H | 300 | 136,196,760,181 | 15,325 |
| Kyrgyzstan | LM | 33 | 8,871,026,074 | 13,047 |
| Lao People's Democratic Republic | LM | 40 | 18,740,559,554 | 18,441 |
| Latvia | H | 75 | 34,343,961,073 | 4,643 |
| Lebanon | UM | 188 | 51,953,744,530 | 15,082 |
| Lesotho | LM | 18 | 2,453,980,835 | 3,324 |
| Liberia | L |  | 3,319,596,500 | 8,916 |
| Libya | UM | 98 | 69,252,306,372 | 18,759 |
| Lithuania | H | 118 | 54,751,510,088 | 6,808 |
| Luxembourg | H | 92 | 69,825,641,851 | 2,074 |
| Madagascar | L |  | 14,104,664,515 | 44,287 |
| Malawi | L |  | 11,025,371,147 | 26,819 |
| Malaysia | UM | 1,872 | 365,175,135,788 | 100,996 |
| Maldives | UM | 24 | 5,609,400,722 | 1,859 |
| Mali | L |  | 17,280,251,194 | 32,074 |
| Malta | H | 32 | 15,725,847,979 | 1,423 |
| Marshall Islands | UM | 3 | 232,092,300 | 140 |
| Mauritania | LM | 16 | 8,066,126,312 | 7,160 |
| Mauritius | H | 60 | 14,436,353,898 | 4,416 |
| Mexico | UM | 3,600 | 1,269,011,767,479 | 325,190 |
| Micronesia (Federated States of) | LM | 2 | 412,000,000 | 252 |
| Monaco | H | 8 | 7,383,745,662 | 132 |
| Mongolia | LM | 23 | 14,206,359,007 | 6,926 |
| Montenegro | UM | 19 | 5,542,053,845 | 1,579 |
| Morocco | LM | 331 | 128,919,944,682 | 89,234 |
| Mozambique | L |  | 15,390,039,242 | 40,768 |
| Myanmar | LM | 385 | 68,697,759,361 | 147,966 |
| Namibia | UM | 50 | 12,541,928,105 | 4,699 |
| Nauru | H | 0 | 118,724,074 | 26 |
| Nepal | LM | 189 | 34,186,190,996 | 76,963 |
| Netherlands | H | 2,371 | 910,194,347,569 | 73,550 |
| New Zealand | H | 835 | 213,434,571,358 | 20,782 |
| Nicaragua | LM | 65 | 12,596,636,042 | 14,387 |
| Niger | L |  | 12,916,455,161 | 30,029 |
| Nigeria | LM | 843 | 448,120,015,210 | 381,113 |
| Niue | 0 |  |  | 5 |
| North Macedonia | UM | 57 | 12,606,338,449 | 5,928 |
| Northern Mariana Islands | H | 1 | 1,182,000,000 | 165 |
| Norway | H | 1,245 | 404,941,363,636 | 17,092 |
| Oman | H | 173 | 88,060,858,257 | 13,046 |
| Pakistan | LM | 1,138 | 320,909,489,230 | 548,187 |
| Palau | H | 2 | 278,900,000 | 63 |
| Palestine | LM | 14 | 17,133,500,000 | 9,971 |
| Panama | H | 177 | 66,984,400,000 | 10,183 |
| Papua New Guinea | LM | 47 | 24,751,344,561 | 22,975 |
| Paraguay | UM | 156 | 37,925,338,329 | 16,249 |
| Peru | UM | 703 | 228,323,495,041 | 85,254 |
| Philippines | LM | 938 | 376,823,278,561 | 298,872 |
| Poland | H | 1,611 | 596,054,591,863 | 105,515 |
| Portugal | H | 759 | 239,986,922,639 | 34,018 |
| Puerto Rico | H | 91 | 105,126,400,000 | 9,161 |
| Qatar | H | 299 | 175,837,550,989 | 11,125 |
| Republic of Moldova | LM | 40 | 11,971,345,003 | 8,954 |
| Romania | H | 662 | 251,019,278,416 | 50,063 |
| Russian Federation | UM | 3,806 | 1,693,113,904,263 | 358,587 |
| Rwanda | L |  | 10,356,327,149 | 22,261 |
| Saint Kitts and Nevis | H | 3 | 1,107,840,741 | 161 |
| Saint Lucia | UM | 4 | 2,094,201,725 | 467 |
| Saint Vincent and the Grenadines | UM | 2 | 910,149,704 | 273 |
| Samoa | UM | 4 | 912,944,538 | 481 |
| San Marino | H | 3 | 1,616,188,702 | 107 |
| Sao Tome and Principe | LM | 1 | 427,425,040 | 410 |
| Saudi Arabia | H | 1,943 | 803,616,264,791 | 114,313 |
| Senegal | LM | 65 | 23,398,811,424 | 26,764 |
| Serbia | UM | 268 | 51,514,222,382 | 23,287 |
| Seychelles | H | 5 | 1,684,373,490 | 353 |
| Sierra Leone | L |  | 4,076,578,543 | 14,064 |
| Singapore | H | 541 | 375,472,731,271 | 19,126 |
| Slovakia | H | 259 | 105,720,354,989 | 14,981 |
| Slovenia | H | 123 | 54,331,588,482 | 5,661 |
| Solomon Islands | LM | 4 | 1,619,154,819 | 1,405 |
| Somalia | L |  | 6,485,000,000 | 25,355 |
| South Africa | UM | 1,575 | 388,531,954,111 | 132,788 |
| South Korea | H | 3,071 | 1,651,422,932,448 | 172,322 |
| South Sudan | L |  |  | 13,869 |
| Spain | H | 3,612 | 1,394,320,055,129 | 151,771 |
| Sri Lanka | LM | 226 | 89,014,990,792 | 67,851 |
| Sudan | L |  | 32,338,079,165 | 76,660 |
| Suriname | UM | 15 | 4,016,040,575 | 1,184 |
| Sweden | H | 2,179 | 533,879,529,188 | 33,054 |
| Switzerland | H | 1,070 | 721,369,112,727 | 29,050 |
| Syrian Arab Republic | L |  | 22,443,299,775 | 31,328 |
| Taiwan (Province of China) | H | 1,534 | 611,396,000,000 | 91,111 |
| Tajikistan | L |  | 8,300,784,857 | 18,129 |
| Thailand | UM | 1,668 | 544,081,056,185 | 233,072 |
| Timor-Leste | LM | 10 | 2,028,551,628 | 2,652 |
| Togo | L |  | 7,220,395,248 | 14,224 |
| Tokelau | 0 |  |  | 4 |
| Tonga | UM | 2 | 512,057,749 | 247 |
| Trinidad and Tobago | H | 61 | 23,849,557,812 | 3,729 |
| Tunisia | LM | 141 | 41,906,113,686 | 31,758 |
| Turkey | UM | 1,870 | 759,937,390,497 | 221,274 |
| Turkmenistan | UM | 107 | 45,231,428,571 | 10,875 |
| Tuvalu | UM | 1 | 54,223,149 | 30 |
| Uganda | L |  | 35,353,060,634 | 56,819 |
| Ukraine | LM | 488 | 153,882,982,016 | 108,429 |
| United Arab Emirates | H | 897 | 417,989,721,734 | 39,072 |
| United Kingdom | H | 4,806 | 2,857,057,847,953 | 164,159 |
| United Republic of Tanzania | LM | 190 | 61,136,873,692 | 89,280 |
| United States of America | H | 66,680 | 21,380,976,119,000 | 1,303,028 |
| Uruguay | H | 213 | 61,231,149,881 | 10,273 |
| Uzbekistan | LM | 232 | 59,907,674,027 | 72,131 |
| Vanuatu | LM | 2 | 936,526,268 | 662 |
| Venezuela (Bolivarian Republic of) | UM | 363 |  | 71,533 |
| Viet Nam | LM | 1,279 | 334,365,257,437 | 346,500 |
| Yemen | L |  |  | 55,800 |
| Zambia | LM | 83 | 23,308,667,781 | 28,887 |
| Zimbabwe | LM | 97 | 21,832,234,926 | 24,145 |

Empty cells indicate that these parameters where not available at the time of analysis.

## Predictors of total costs: Multiple linear regression results

| Predictor | β | SE | t | p | 95% CI |
| --- | --- | --- | --- | --- | --- |
| GDP | 0.967 | 0.009 | 106.04 | <.001 | [0.949, 0.985] |
| Prevalence cases | 0.043 | 0.009 | 4.73 | <.001 | [0.025, 0.061] |
